# A bacterial chemoreceptor that mediates chemotaxis to two different plant hormones

**DOI:** 10.1111/1462-2920.15920

**Published:** 2022-02-01

**Authors:** Miriam Rico‐Jiménez, Amalia Roca, Tino Krell, Miguel A. Matilla

**Affiliations:** ^1^ Department of Environmental Protection, Estación Experimental del Zaidín, Consejo Superior de Investigaciones Científicas Granada Spain; ^2^ Department of Microbiology, Facultad de Farmacia Campus Universitario de Cartuja, Universidad de Granada Granada 18071 Spain

## Abstract

Indole‐3‐acetic acid (IAA) is the main naturally occurring auxin and is produced by organisms of all kingdoms of life. In addition to the regulation of plant growth and development, IAA plays an important role in the interaction between plants and growth‐promoting and phytopathogenic bacteria by regulating bacterial gene expression and physiology. We show here that an IAA metabolizing plant‐associated *Pseudomonas putida* isolate exhibits chemotaxis to IAA that is independent of auxin metabolism. We found that IAA chemotaxis is based on the activity of the PcpI chemoreceptor and heterologous expression of *pcpI* conferred IAA taxis to different environmental and human pathogenic isolates of the *Pseudomonas* genus. Using ligand screening, microcalorimetry and quantitative chemotaxis assays, we found that PcpI failed to bind IAA directly, but recognized and mediated chemoattractions to various aromatic compounds, including the phytohormone salicylic acid. The expression of *pcpI* and its role in the interactions with plants was also investigated. PcpI extends the range of central signal molecules recognized by chemoreceptors. To our knowledge, this is the first report on a bacterial receptor that responds to two different phytohormones. Our study reinforces the multifunctional role of IAA and salicylic acid as intra‐ and inter‐kingdom signal molecules.

## Introduction

The phytohormone indole‐3‐acetic acid (IAA) is the most common naturally occurring auxin and is key for plant growth, development and defence, playing essential roles in embryogenesis, *de novo* organogenesis, vascular formation as well as seed, root and flower development, among other processes (Zhao, 2018; Gallei *et al*., 2020). However, IAA is an ubiquitous signalling molecule, since bacteria (Kunkel and Harper, 2018; Duca and Glick, 2020), fungi (Fu *et al*., 2015), archaea (Aklujkar *et al*., 2014), algae (Bogaert *et al*., 2019; Laird *et al*., 2020) and animals (Oliveira *et al*., 2007) were found to produce IAA. This ubiquity, together with a growing body of experimental evidence, supports the role of IAA as an inter‐ and intra‐kingdom signal molecule. For example, IAA was found to regulate cell division and development in algae (Ohtaka *et al*., 2017; Bogaert *et al*., 2019) and IAA produced by various algae modulate different virulence traits in an aquatic bacterial pathogen (Yang *et al*., 2017). Alternatively, bacteria co‐occurring with marine diatoms were shown to promote diatom growth through the synthesis of IAA (Amin *et al*., 2015). In fungi, IAA affected growth, sporulation, spore germination as well as fungal competitiveness (Fu *et al*., 2015; Liu *et al*., 2016; Nicastro *et al*., 2021) and fungal IAA synthesis modulated growth, development and immune responses in plant hosts (Fu *et al*., 2015; Jahn *et al*., 2021).

Many plant‐associated bacteria synthesize IAA (Spaepen and Vanderleyden, 2011; Duca *et al*., 2014; Kunkel and Harper, 2018; Duca and Glick, 2020), which has been shown to play crucial roles during their interaction with their hosts. Indeed, IAA production was found to be involved in nodule formation and nitrogen fixation by rhizobia in legume plants as well as in the stimulation of plant growth by non‐symbiotic beneficial rhizobacteria (Spaepen and Vanderleyden, 2011; Duca and Glick, 2020). Furthermore, IAA plays an essential role in plant–phytobacteria interactions, typically promoting plant susceptibility and disease development by different mechanisms that include the alteration of the IAA balance in the plant, the suppression of host basal defence responses and the regulation of the synthesis of virulence factors in the bacterial pathogen (Kunkel and Johnson, 2021). Beyond the role of bacterial IAA in the interaction with plants, a number of studies have provided first insight into the molecular basis of IAA action in phytobacteria, as it was shown to modulate gene expression and numerous physiological processes such as stress tolerance, primary metabolism, production of virulence factors, antibiotic synthesis and biofilm formation (Duca *et al*., 2014; Kunkel and Harper, 2018; Matilla *et al*., 2018; Duca and Glick, 2020; Djami‐Tchatchou *et al*., 2021). In addition, there is also growing evidence for a role of IAA in the modulation of bacterial motility and chemotaxis in plant‐associated bacteria like *Rhizobium etli* (Spaepen *et al*., 2009), *Bradyrhizobium japonicum* (Donati *et al*., 2013) and *Pseudomonas syringae* (Soby *et al*., 1991; Djami‐Tchatchou *et al*., 2021). However, the molecular mechanisms behind most of these IAA‐mediated processes remain unknown.

Chemotaxis permits bacteria to adapt their swimming motility patterns in chemical gradients, thus favouring access to nutritional sources and preferred environments for growth (Matilla and Krell, 2018; Colin *et al*., 2021). Typically, chemotaxis signalling is initiated by the recognition of chemoeffectors by the ligand‐binding domain (LBD) of a chemoreceptor. Chemoeffector binding causes a molecular stimulus that modulates the autophosphorylation activity of the histidine kinase CheA, subsequently altering the transphosphorylation activity of the response regulator CheY. Phosphorylated CheY binds to the flagellar motor resulting in a change in the direction of flagellar rotation, ultimately causing a chemotactic response (Bi and Sourjik, 2018; Matilla *et al*., 2021a). To date, most chemoeffectors identified appear to be compounds of metabolic value such as sugars, amino acids and organic acids that can serve as nutrient and energy sources for bacteria (Sampedro *et al*., 2015; Matilla *et al*., 2021a; Matilla *et al*., 2022). However, other chemoeffectors like animal (Lopes and Sourjik, 2018) and plant (Kim *et al*., 2007; Antunez‐Lamas *et al*., 2009) hormones, quorum sensing molecules (Zhang *et al*., 2020), plant defence metabolites (Neal *et al*., 2012) and neurotransmitters (Pasupuleti *et al*., 2014; Corral‐Lugo *et al*., 2018) can alternatively provide information about favourable, and even highly specific, environmental niches.

The plant‐associated bacterium *Pseudomonas putida* 1290 was isolated from a pear plant due to its ability to efficiently use IAA as carbon, nitrogen and energy source (Leveau and Lindow, 2005). Indeed, *P*. *putida* 1290 was the first bacterium for which the gene cluster responsible for IAA degradation, named *iacABCDEFGRHI*, was described (Leveau and Gerards, 2008), and it is currently used as a model for the isolation and characterization of genes involved in IAA degradation (Laird *et al*., 2020). Significantly, the IAA catabolic properties of *P*. *putida* 1290 were shown to alleviate the detrimental effects that appear on plants caused by the exogenous addition of IAA (Leveau and Gerards, 2008) or due to the production of high levels of IAA by rhizosphere microbial competitors (Leveau and Lindow, 2005). Using swim plate motility assays, *P*. *putida* 1290 was shown to exhibit directed movement towards IAA (Scott *et al*., 2013). However, such plate‐based assays do not permit to distinguish between chemotaxis and energy taxis; a lower specific form of directed cell movement to environmental sites at which the cellular metabolism is optimal (Schweinitzer and Josenhans, 2010; Colin *et al*., 2021). Heterologous expression of the *iac* catabolic cluster in *P*. *putida* KT2440 provided this bacterium with the ability to use IAA as nutrient and energy source but did not confer the ability to migrate towards IAA, as determined by swim plate assays (Scott *et al*., 2013) – suggesting that a specific IAA chemoreceptor encoded in the genome of *P*. *putida* 1290 may be responsible for the observed behaviour. To our knowledge, no evidence of IAA chemotaxis has been reported in other bacterial strains.

We show here that IAA chemotaxis in *P*. *putida* 1290 is based on the action of the chemoreceptor PcpI that employs a mechanism that does not involve energy taxis. PcpI was also found to mediate taxis to additional chemoeffectors, including the phytohormone salicylic acid. The expression of *pcpI* and its role in plant root colonization was also studied. This work expands the range of chemoreceptors that are stimulated by important signal molecules of life.

## Results

### IAA chemotaxis of *Pseudomonas putida* 1290 does not dependent on auxin metabolism

**Fig. 1:**
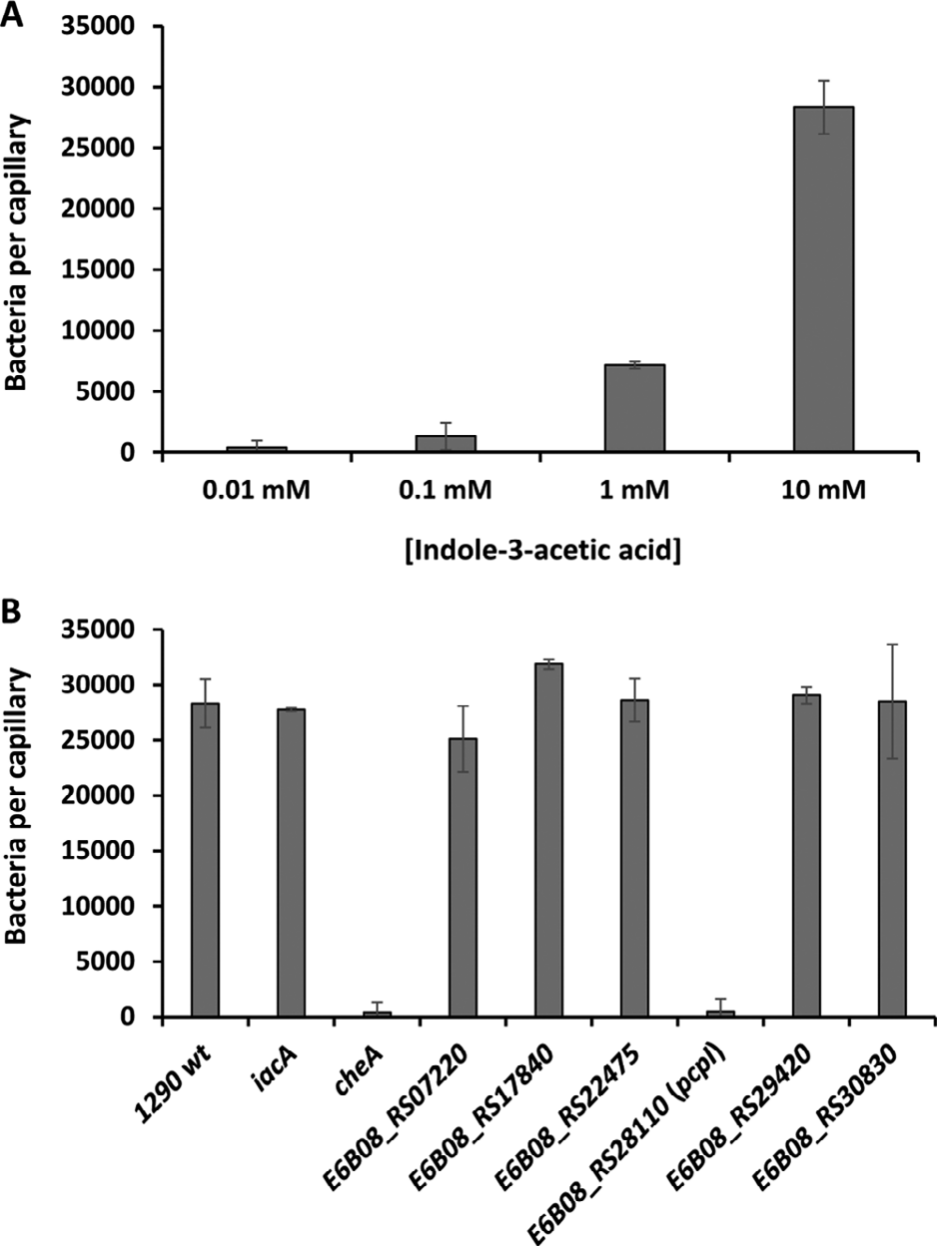
Chemotaxis of *Pseudomonas putida* 1290 wild type and mutant strains towards indole‐3‐acetic acid (IAA). A. Quantitative capillary chemotaxis assays of the wild type strain to different concentrations of IAA. B. Chemotaxis to 10 mM IAA of different strains of *P*. *putida* 1290. In all cases, data were corrected with the number of cells that swam into buffer containing capillaries. Shown data are means and standard deviations from three independent experiments conducted in triplicate.

To investigate the chemotactic behaviour of *P*. *putida* 1290 towards IAA, we conducted quantitative capillary chemotaxis assays – an experimental approach that primarily monitors chemotaxis and to a much lower degree energy taxis. IAA was tested at concentrations ranging from 0.01 to 10 mM, with optimal chemotactic responses at 10 mM and an onset at 100 μM of IAA (Fig. 1A). These concentrations do not necessarily account for the minimum threshold for chemotaxis since the chemoeffector concentration decreases sharply from the capillary source (Raina *et al*., 2019; Tunchai *et al*., 2021). In analogy to *P*. *putida* KT2440 (Lopez‐Farfan *et al*., 2019), *P*. *putida* 1290 has three chemosensory pathways and a mutant defective in *cheA*, present within the chemotaxis signalling gene cluster, failed to respond to IAA (Fig. 1B).

In order to rule out the involvement of IAA metabolism in the observed chemotactic response, we generated a polar mutant in the first gene of the IAA catabolic operon, *iacA* (Leveau and Gerards, 2008). Mutation of *iacA* resulted in the inability to grow on IAA as sole carbon source (Supp. Figs. S1A and S2) and this mutant strain showed wild type like chemotaxis towards IAA using quantitative capillary chemotaxis assays (Fig. 1B) – confirming that the observed response is not based on energy taxis.

### The chemoreceptor repertoire of *P*. *putida* 1290

**Fig. 2:**
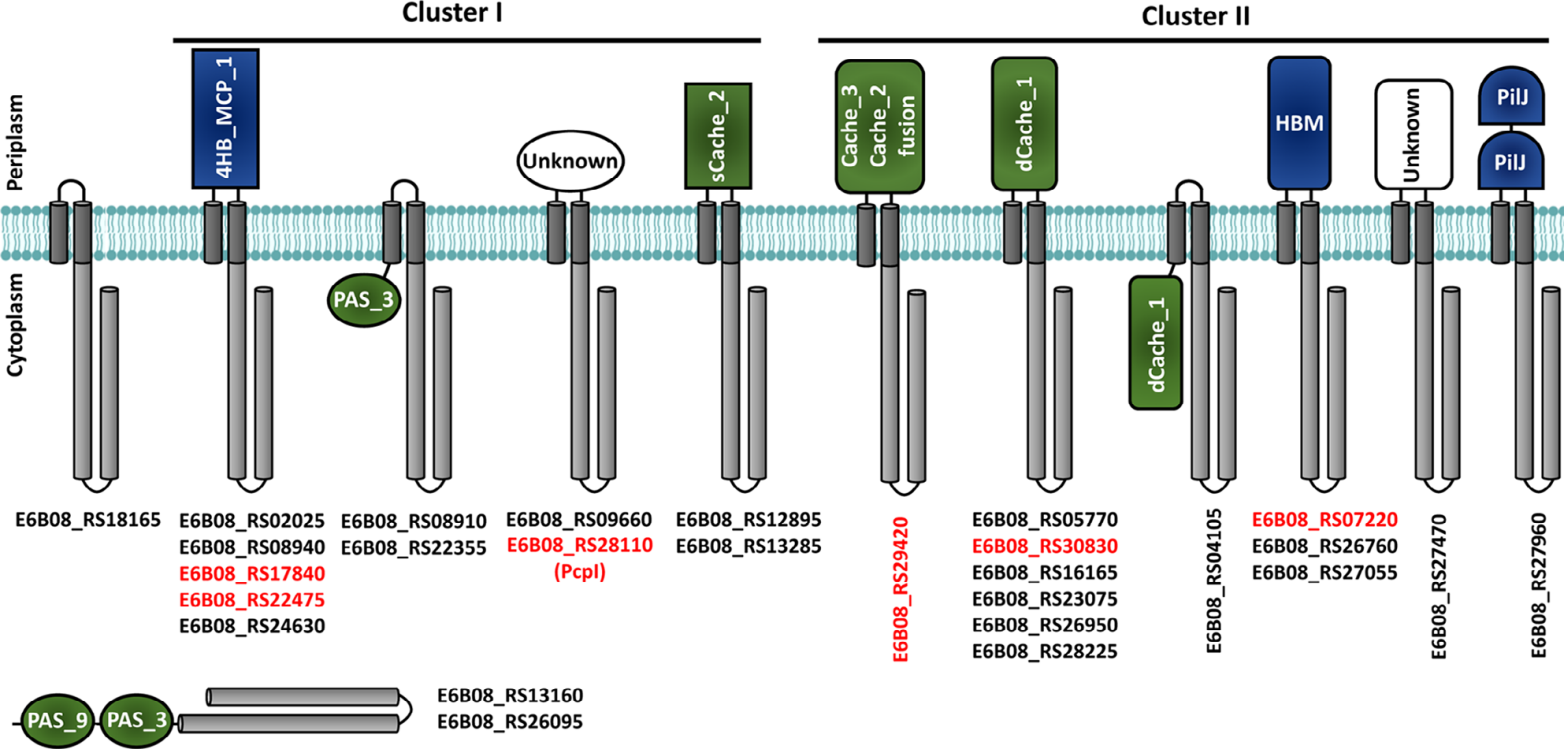
The chemoreceptor repertoire of *Pseudomonas putida* 1290. Predicted receptor topology and locus tags are shown. Annotation was based on the Pfam database and, in case of un‐annotated LBDs, domain type was defined by visual inspection of homology models generated by the Phyre2 algorithm (Kelley *et al*., 2015). Topologies are based on the prediction of transmembrane regions using the DAS algorithm (Cserzo *et al*., 1997). Chemoreceptors were organized into cluster I and cluster II based on the length of their LBDs, as described previously (Lacal *et al*., 2010b). Ligand binding domains with α/β folds or parallel helices are shown in green and blue, respectively. Chemoreceptor names in red indicate receptors that do not have homologues in *P*. *putida* KT2440 and *P*. *aeruginosa* PAO1 (i.e. LBDs with <41% sequence identity). 4‐HB, 4‐helix bundle domain; HBM, helical bimodular domain; PAS, Per‐Arnt‐Sim domain; PilJ, Type IV pili domain; Unknown, LBDs of unknown type.

The genome of *P*. *putida* 1290 (Laird and Leveau, 2019) encodes 27 chemoreceptors (Fig. 2), which corresponds or is similar to the number of chemoreceptors encoded in two *Pseudomonas* chemotaxis model strains, namely *P*. *putida* KT2440 (Lopez‐Farfan *et al*., 2019) and *P*. *aeruginosa* PAO1 (26 chemoreceptors) (Matilla *et al*., 2021a), respectively. At least 10 different types of LBDs were identified in *P*. *putida* 1290 chemoreceptors, including LBDs consisting of parallel helices (e.g. 4HB_MCP‐1, HBM, PilJ) and α/β folds (e.g. sCache_2, dCache_1, Cache_3‐Cache_2, PAS_3, PAS_9) (Fig. 2). The most abundant LBD types were dCache_1 and 4HB_MCP_1, which are also the most abundant LBDs in bacterial chemoreceptors (Upadhyay *et al*., 2016; Ortega *et al*., 2017). Twenty‐one (i.e. 78%) of the *P*. *putida* 1290 chemoreceptors showed the canonical topology and are transmembrane proteins with their LBD located in the periplasm. However, the structural and topological diversity of *P*. *putida* 1290 chemoreceptors was reflected by the presence of a transmembrane chemoreceptor that lacks an LBD, three membrane‐associated receptors with cytosolic PAS_3 and dCache_1 LBDs as well as two entirely cytosolic receptors composed of two tandem PAS domains (Fig. 2). The latter two receptors are likely to be involved in the sensing of cytosolic signals like redox‐active cofactors or oxygen (Collins *et al*., 2014).

### Identification of PcpI as the chemoreceptor responsible for IAA chemotaxis

Around half of the chemoreceptors of KT2440 and PAO1 have been characterized and some of their ligands include amino acids, organic acids, phytohormones, polyamines and inorganic nutrients, among others (Ortega *et al*., 2017; Matilla *et al*., 2021a). Quantitative capillary chemotaxis assays of KT2440 and PAO1 showed that both strains failed to respond to different concentrations of IAA (Supp. Fig. S3). Based on these results, we hypothesized that a receptor that was absent in KT2440 and PAO1 would be responsible for IAA taxis in *P*. *putida* 1290.

The ligand specificity of most chemoreceptors is determined by their rapidly evolving LBDs (Ortega *et al*., 2017; Gavira *et al*., 2020; Matilla *et al*., 2021a). None of the 27 chemoreceptors of *P*. *putida* 1290 has been characterized and to identify the IAA chemoreceptor, we performed homology comparisons between LBD sequences of *P*. *putida* 1290 chemoreceptors with those of KT2440 and PAO1. These analyses revealed that *P*. *putida* 1290 has 19 and 15 chemoreceptors that are homologous (i.e. LBDs with more than 41% sequence identity) to the receptors present in KT2440 and PAO1 respectively (Table 1). Homologous chemoreceptors were found to mediate taxis towards amino acids (e.g. PctA, PctC, McpA), organic acids (e.g. McpR, McpP, McpS, PA2652), polyamines (e.g. TlpQ, McpU) and inorganic phosphate (Pi) (e.g. CtpH, CtpL). Furthermore, a receptor homologous to the energy taxis chemoreceptor Aer or to proteins that mediate alternative cellular functions such as the modulation of intracellular levels of second messengers (e.g. WspA, PilJ, BdlA) were also found (Table 1). Notably, we identified six *P*. *putida* 1290 chemoreceptors that were either not present in KT2440 or PAO1 (e.g. E6B08_RS07220, E6B08_RS17840, E6B08_RS22475, E6B08_RS28110, E6B08_RS29420) or which LBD had low level of sequence identity (e.g. E6B08_RS30830). These chemoreceptors have different types of LBDs, including 4HB_MCP_1, sCache_3‐sCache_2, dCache_1 and HBM (Fig. 2; Table 1).

**Table 1:** *Pseudomonas putida* 1290 chemoreceptors and their characterized homologues of *P*. *putida* KT2440 and *P*. *aeruginosa* PAO1.

Chemoreceptor	LBD name (Pfam)	Closest homologue in KT2440 (% identity)	Closest homologue in PAO1 (% identity)	Chemoeffector(s)/comment(s)	Reference(s)
E6B08_RS02025	4HB_MCP_1 (PF12729)	PP_0317/McpR (67.3%)	–	Succinate, malate, fumarate	Parales *et al*. (2013)
E6B08_RS04105	Not annotated (dCache_1‐like)^a^	PP_3950 (76.4%)	–	Unknown	–
E6B08_RS05770	dCache_1 (PF02743)	PP_2249/McpA (45.1%)	PA4309/PctA (56.0%)	Amino acids	Rico‐Jimenez *et al*. (2013), Corral‐Lugo *et al*. (2016), Gavira *et al*. (2020)
E6B08_RS07220	HBM (PF16591)	–	–	Unknown	–
E6B08_RS08910	PAS_3 (PF08447)	PP_2111/Aer2 (89.5%)	PA1561/Aer/TlpC (76.5%)	Energy taxis	Hong *et al*. (2004a), Hong *et al*. (2004b), Sarand *et al*. (2008)
E6B08_RS08940	Not annotated (4HB_MCP_1‐like)^a^	PP_2120/CtpH_PP (82.4%)	PA2561/CtpH (50.6%)	Inorganic phosphate	Wu *et al*. (2000), Rico‐Jimenez *et al*. (2016)
E6B08_RS09660	Small unknown	PP_2310 (68.2%)	PA2867 (40.5%)	Mutation in *PP2310* increases biofilm formation	Corral‐Lugo *et al*. (2016)
E6B08_RS12895	sCache_2 (PF17200)	–	PA2652 (45.2%)	l‐malate, bromosuccinate, citramalate	Martin‐Mora *et al*. (2018b)
E6B08_RS13160	PAS_9‐PAS_3 (PF13426–PF08447)	PP_3414/Aer (71.4%)	BldA (51.3%)	BdlA is involved in biofilm dispersion	Morgan *et al*. (2006), Petrova and Sauer (2012b), Petrova and Sauer (2012a)
E6B08_RS13285	sCache_2 (PF17200)	PP_2861/McpP (88.1%)	–	Pyruvate, l‐lactate, propionate, acetate	Garcia *et al*. (2015)
E6B08_RS16165	dCache_1 (PF02743)	PP_3557 (80.2%)	PA2654/TlpQ (53.4%)	Polyamines	Corral‐Lugo *et al*. (2018)
E6B08_RS17840	4HB_MCP_1 (PF12729)	–	–	Unknown	–
E6B08_RS18165	No LBD	–	–	Unknown	–
E6B08_RS22355	PAS_3 (PF08447)	PP_4521/Aer3 (81.4%)	PA1561/Aer (60.8%)	Energy taxis?	Sarand *et al*. (2008)
E6B08_RS22475	4HB_MCP_1 (PF12729)	–	–	Unknown	–
E6B08_RS23075	dCache_1 (PF02743)	PP_1228/McpU (76.8%)	PA2654/TlpQ (49.0%)	Polyamines	Corral‐Lugo *et al*. (2016), Corral‐Lugo *et al*. (2018)
E6B08_RS24630	4HB_MCP_1 (PF12729)	PP_1488/WspA_PP (68.1%)	PA3708/WspA (36.7%)	Surface sensing, modulation of c‐di‐GMP levels	O'Connor *et al*. (2012), Chen *et al*. (2014), Corral‐Lugo *et al*. (2016)
E6B08_RS26095	PAS_9‐PAS_3 (PF13426‐PF08447)	PP_0779 (72.3%)	BldA (38.3%)	BdlA is involved in biofilm dispersion	Morgan *et al*. (2006), Petrova and Sauer (2012b), Petrova and Sauer (2012a)
E6B08_RS26760	HBM (PF16591)	PP_4658/McpS (73.6%)	–	Malate, fumarate, oxaloacetate, succinate, citrate, isocitrate , butyrate	Lacal *et al*. (2010a), ), Pineda‐Molina *et al*. (2012)
E6B08_RS26950	dCache_1 (PF02743)	PP_0584 /McpC (82.9%)	–	Cytosine?, nicotinic acid?	Liu *et al*. (2009), Parales *et al*. (2014)
E6B08_RS27055	Not annotated (HBM‐like)^a^	PP_0562/CtpL_PP (82.5%)	PA4844/CtpL (55.6%)	Inorganic phosphate	Wu *et al*. (2000), Rico‐Jimenez *et al*. (2016)
E6B08_RS27470	Large unknown	PP_4888 (84.9%)	–	Expression regulated by benzoxazinoids	Neal *et al*. (2012)
E6B08_RS27960	PilJ‐PilJ (PF13675)	PP_4989/PilJ (93.4%)	PA0411/PilJ (73.5%)	Surface sensing, modulation of c‐di‐GMP and cAMP levels	Fulcher *et al*. (2010), Luo *et al*. (2015), Jansari *et al*. (2016)
E6B08_RS28110 (PcpI)	Small unknown	–	–	IAA, salicylate, benzoate, 3‐methylbenzoate	This study
E6B08_RS28225	dCache_1 (PF02743)	PP_2249/McpA (40.7%)	PA4307/PctC (43.5%)	Amino acids	Rico‐Jimenez *et al*. (2013), Corral‐Lugo *et al*. (2016), Gavira *et al*. (2020)
E6B08_RS29420	Cache_3‐Cache_2 (PF17201)	–	–	Unknown	–
E6B08_RS30830	dCache_1 (PF02743)	PP_1228/McpU (38.3%)	PA2654/TlpQ (40.2%)	Polyamines	Corral‐Lugo *et al*. (2016), Corral‐Lugo *et al*. (2018)

^a^ Domain type un‐annotated in Pfam and defined by visual inspection of a homology model generated using the Phyre2 algorithm (Kelley *et al*., 2015).

To assess the potential involvement of these receptors in IAA chemotaxis of *P*. *putida* 1290, we constructed mutants in the corresponding genes which were subsequently phenotypically characterized using quantitative capillary chemotaxis assays. We found that the mutant defective in the E6B08_RS28110 chemoreceptor was the only strain that showed no chemotaxis to IAA (Fig. 1B) – a tactic phenotype that was indistinguishable to that of a mutant defective in *cheA* (Fig. 1B). Control experiments showed that the *E6B08_RS28110* mutant showed wild type like chemotaxis to casamino acids (Supp. Fig. S4), indicating that the *E6B08_RS28110* mutation does not cause a general chemotactic defect. Swim plate chemotaxis assays containing IAA as sole carbon source revealed only a slight decrease in the motility of the *E6B08_RS28110* mutant compared to the parental strain (Supp. Fig. S2) – supporting that IAA energy taxis masks to a large degree IAA chemotaxis and that the initial tactic phenotype observed in swim plate assays (Scott *et al*., 2013) was primarily driven by energy taxis. As observed here, energy taxis was previously shown to mask chemotaxis using swim plate assays (Alvarez‐Ortega and Harwood, 2007; Parales *et al*., 2013).

**Fig. 3:**
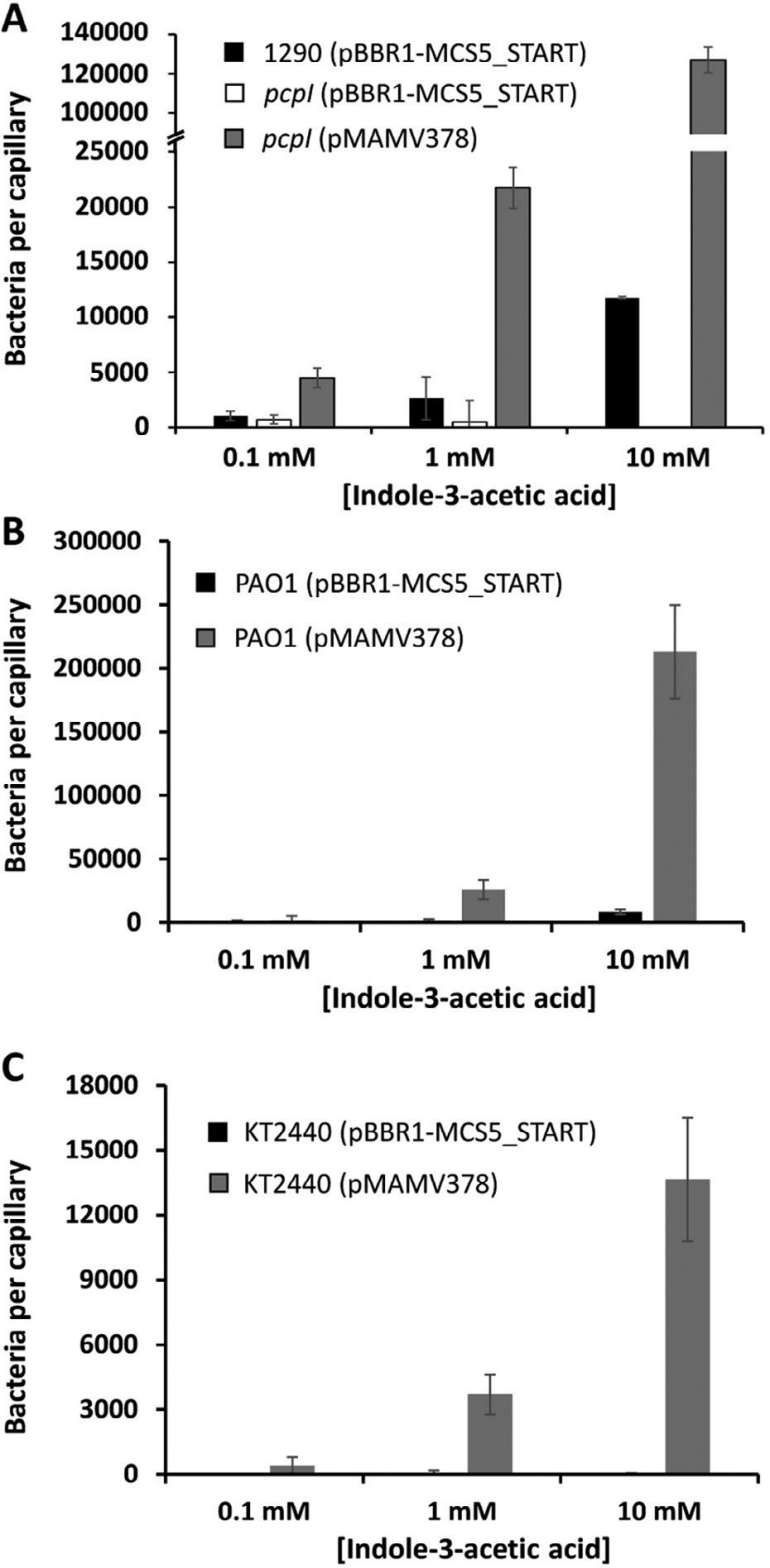
*In trans* expression of *pcpI* in different *Pseudomonas* strains. Multicopy expression of *pcpI* from the pBBR1‐MCS5_START derivative plasmid pMAMV378 increases the magnitude of IAA chemotaxis in *P*. *putida* 1290 (A) and confers IAA taxis to *P*. *aeruginosa* PAO1 (B) and *P*. *putida* KT2440 (C). Data are means and standard deviations from three independent experiments conducted in triplicate.

To confirm the association between the *E6B08_RS28110* mutation and the loss of IAA chemotaxis, we cloned the *E6B08_RS28110* gene into a pBBR1MCS‐based medium copy number plasmid. *In trans* expression of *E6B08_RS28110* not only restored chemotaxis to IAA in the mutant strain but also increased the magnitude of chemotaxis more than 10‐fold compared to the wild type strain (Fig. 3A). These results imply that enhanced cellular chemoreceptor levels were responsible for an increased chemotactic behaviour towards IAA, as described previously for other chemoreceptors (Fernández *et al*., 2016; Hida *et al*., 2020). To determine whether E6B08_RS28110 can confer the IAA chemotaxis phenotype to KT2440 and PAO1, we expressed heterologously the *E6B08_RS28110* gene in these bacterial strains. We found that E6B08_RS28110 conferred IAA chemotaxis to both strains (Fig. 3B and C), inducing a particularly strong response in PAO1 (Fig. 3B). Based on these results, the chemoreceptor E6B08_RS28110 was named PcpI (*Pseudomonas* chemoreceptor protein IAA).

### Expression of *pcpI* correlates with the magnitude of IAA chemotaxis

**Fig. 4:**
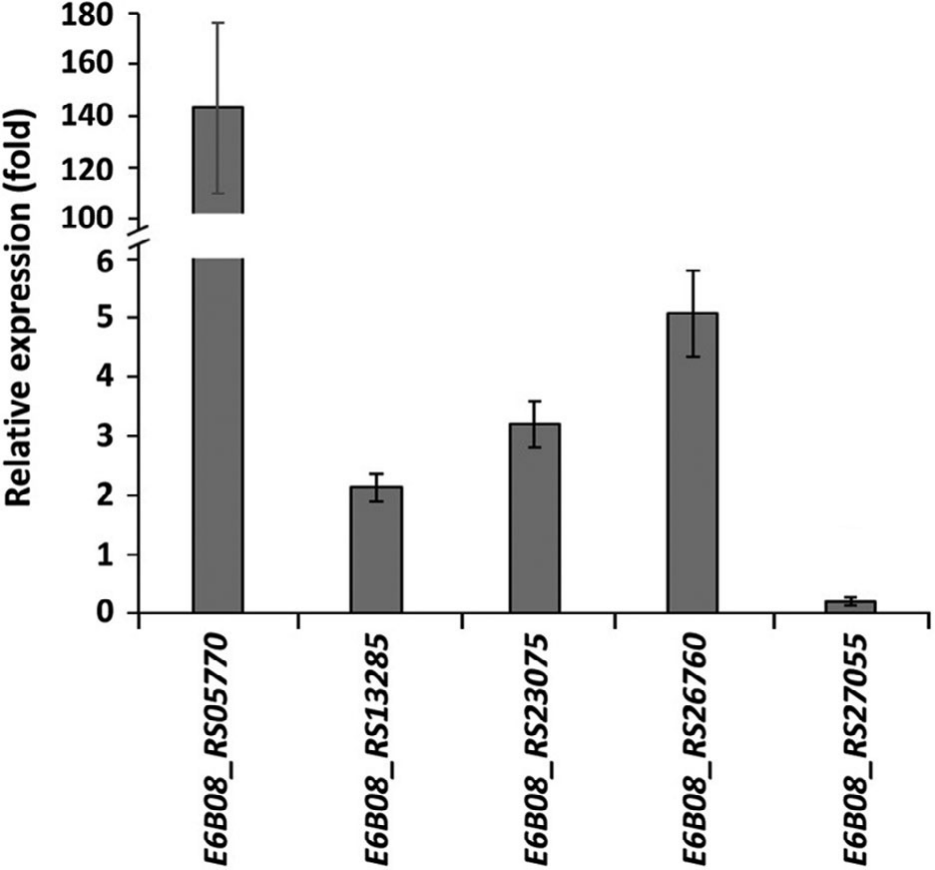
Transcript levels of *P*. *putida* 1290 chemoreceptor genes in comparison to transcript levels of *pcpI* measured by quantitative real‐time PCR. The values showed the expression of five chemoreceptor genes relative to *pcpI* expression. Data are the means and standard deviations from three biological replicates conducted in triplicate.

The observation that multicopy expression of the *pcpI* gene dramatically increased chemotactic responses towards IAA encouraged us to investigate the expression of *pcpI* in comparison with other chemoreceptor genes present in the genome of 1290. Since we found in *P*. *putida* 1290 homologous chemoreceptors that respond to amino acids, polyamines, organic acids and Pi (Table 1), we first conducted chemotaxis assays to 1 mM concentrations of arginine, putrescine, propionate, oxaloacetate and Pi. Quantitative chemotaxis assays revealed that *P*. *putida* 1290 showed strong chemotactic responses to polyamines, amino and organic acids (Supp. Fig. S5), whereas only minor responses to Pi were observed (Supp. Fig. S5), which may be due to the low expression of the corresponding chemoreceptor genes under conditions of Pi excess (Wu *et al*., 2000; Bains *et al*., 2012). We subsequently analyzed the transcript levels of *pcpI* under the same growth conditions used to conduct chemotaxis assays, namely mid‐logarithmic growth phase in M9 minimal medium supplemented glucose as carbon source, and compared these to the transcript levels of the chemoreceptor genes homologous to receptors involved in amino acid, organic acid, polyamine and Pi chemotaxis in another model *Pseudomonas*. The results showed that *pcpI* transcript levels were between 2.1‐ and 143.2‐fold lower than those of *E6B08_RS05770*, *E6B08_RS13285*, *E6B08_RS23075* and *E6B08_RS26760* (Fig. 4) – chemoreceptor genes homologous to *pctA*, *mcpP*, *mcpU* and *mcpS,* respectively (Table 1). In contrast, the expression of *pcpI* was 5.0 times higher than *E6B08_RS27055*, a *ctpL* homologue, which is in accordance with the very low chemotactic responses to Pi (Supp. Fig. S5). Taken together, these results correlate *pcpI* expression with the chemotactic responses observed towards IAA.

### PcpI does not recognize IAA directly but the phytohormone salicylic acid

**Fig. 5:**
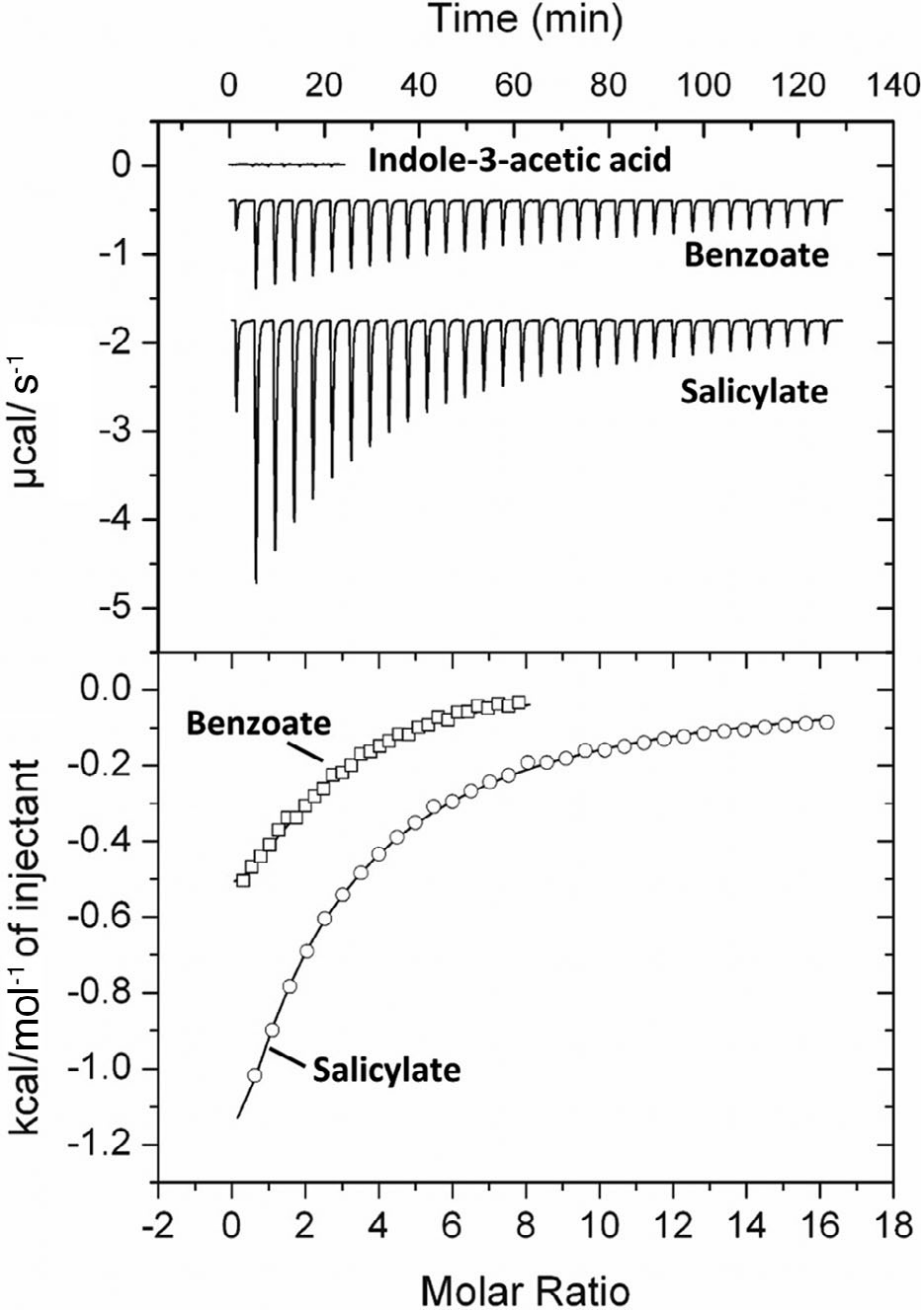
Isothermal titration calorimetry analysis of ligand binding to PcpI‐LBD. Upper panel: Raw data for the titration of PcpI‐LBD with 9.6 μl aliquots of indole‐3‐acetic acid (3 mM), salicylate (2 mM) and benzoate (5 mM). Lower panel: Integrated, dilution heat‐corrected and concentration‐normalized peak areas of the titration data for PcpI‐LBD. Data were fitted using the ‘one binding site’ model of the MicroCal version of ORIGIN. The derived thermodynamic parameters are provided in Suppl. Table S1.

To delve into the molecular mechanisms of IAA chemotaxis in *P*. *putida* 1290, we cloned the DNA fragment encoding the LBD of PcpI into an expression vector and purified the protein by affinity chromatography. Subsequently, recombinant PcpI‐LBD was submitted to microcalorimetric titrations with IAA. We did not observe binding heats in microcalorimetric titrations conducted at two different temperatures, 25°C and 10°C, indicative of an absence of binding (Fig. 5). To assess the possibility that PcpI may be stimulated by the binding of an IAA‐loaded solute binding protein (SBP), we conducted pulldown assays with immobilized PcpI‐LBD and *P*. *putida* 1290 protein extracts but found no evidence for an SBP involved (Supp. Fig. S6).

Typically, SBPs that interact with chemoreceptors are encoded in transporter gene clusters (Matilla *et al*., 2021b). Genome analysis of *P*. *putida* 1290 revealed the presence of an ABC type transporter gene cluster, E6B08_RS28115‐E6B08_RS28125, immediately downstream of *pcpI*. The TransportDB database (Elbourne *et al*., 2017) predicted this ABC transporter to be involved in the uptake of amino acids. Given that there are transcriptional regulators (Marmorstein and Sigler, 1989; Herud‐Sikimić *et al*., 2021) as well as SBPs (Vetting *et al*., 2015) that bind both, amino acids and IAA, we purified the SBP of this transporter, E6B08_RS28125, and isothermal titration calorimetry (ITC) assays with IAA revealed no binding (Supp. Fig. S7). Subsequently, we used differential scanning fluorimetry (DSF) (Martin‐Mora *et al*., 2018a) and microcalorimetric titrations to analyze the ligand of profile of E6B08_RS28125 and found that E6B08_RS28125 binds l‐ornithine, l‐His and l‐Arg with dissociation constants (*K*_D_) of 0.9 ± 0.1, 3.3 ± 0.3 and 29.5 ± 3 μM, respectively (Supp. Figs S7 and S8; Supp. Table S1). Further protein–protein interaction assays using ITC revealed no evidence of protein complex formation between PcpI‐LBD and E6B08_RS28125 (Supp. Fig. S9).

To identify ligands that are directly recognized by PcpI, the LBD of PcpI was submitted to high‐throughput ligand screening using DSF. We screened ~480 compounds from the Biolog Compound arrays PM1, PM2A, PM3B, PM4A and PM5 that contain multiple carbon, nitrogen, sulfur and phosphorus sources. We found that ligand‐free PcpI‐LBD has a midpoint of protein unfolding transition (Tm) of 39.6°C and that salicylate caused an increase in the Tm of PcpI‐LBD of 2.6°C (Supp. Fig. S10). No additional compounds causing Tm shifts were identified. To confirm binding, PcpI‐LBD was titrated with salicylate. Exothermic heats were observed that decreased as protein saturation progressed and a *K*_D_ of 826 ± 34 μM was derived (Fig. 5; Supp. Table S1). We subsequently analyzed 14 additional aromatic and non‐aromatic C6‐ring containing molecules (listed in the legend to Supp. Table S1) and found binding for benzoate and 3‐methylbenzoate (3‐MBA) with affinities of 171 ± 14 and 91 ± 8 μM, respectively (Fig. 5; Supp. Table S1). We therefore conclude that PcpI directly binds the carboxylic acid aromatic compounds salicylate, benzoate and 3‐MBA.

### PcpI mediates chemotaxis to benzoate, 3‐MBA and salicylate

**Fig. 6:**
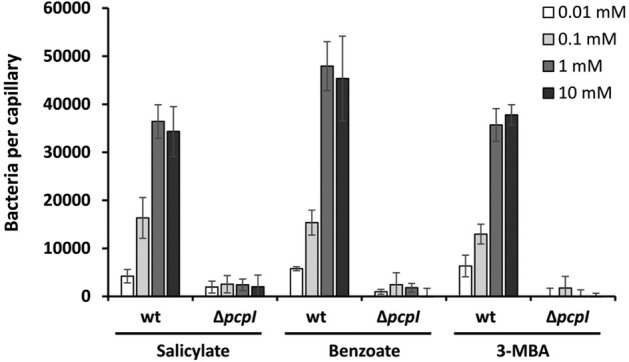
Quantitative capillary chemotaxis assays of *Pseudomonas putida* 1290 wild type and a *pcpI* mutant to different carboxylic acid aromatic ligands of PcpI. In all cases, data were corrected with the number of cells that swam into buffer containing capillaries. Shown data are means and standard deviations from three independent experiments conducted in triplicate. 3‐MBA, 3‐methylbenzoate.

To assess the relevance of benzoate, 3‐MBA and salicylate on the physiology of *P*. *putida* 1290, we first conducted quantitative capillary assays. The strain 1290 exhibited chemotaxis towards the three ligands with an onset of chemotaxis at 10 μM and a maximal response at 1 mM for all three compounds (Fig. 6). The magnitude of the response was similar for the three PcpI ligands, although a slightly greater tactic response was observed for benzoate at concentrations above 1 mM (Fig. 6). Contrary to what was previously observed for other chemoreceptors (Reyes‐Darias *et al*., 2015; Fernandez *et al*., 2017), no correlation was observed between the affinity of the chemoreceptor LBD for the ligands and the magnitude of the chemotactic response. The *in vivo* response occurred at concentrations well below the *K*_D_ for ligand recognition (Figs. 5 and 6).

To determine the role of PcpI in the observed tactic responses to aromatic compounds, quantitative capillary assays with a mutant defective in *pcpI* were carried out. The results showed that the deletion of *pcpI* caused the complete disappearance of chemotaxis to all three ligands over the entire concentration range (Fig. 6), indicating that PcpI is the sole *P*. *putida* 1290 chemoreceptor for benzoate, 3‐MBA and salicylate under the conditions tested.

We subsequently analyzed the metabolic relevance of the three PcpI ligands by conducting growth experiments in minimal medium containing each of the chemoattractants as sole carbon source. We found that benzoate and salicylate served as growth substrates for *P*. *putida* 1290 (Supp. Fig. S1B,C), whereas 3‐MBA did not support the growth of strain 1290 (Supp. Fig. S1D).

### Role of PcpI in the chemotaxis towards root exudates and plant colonization

To evaluate the relevance of PcpI for establishing interactions with plants, we conducted competitive root colonization assays. In these assays, *P*. *putida* 1290 wild type and a *pcpI* mutant were inoculated at a certain distance from the maize seedlings and the number of wild type and mutant bacteria that colonized the roots 10 days post‐inoculation were quantified. We determined that *P*. *putida* 1290 colonizes maize roots at a density of around 7 × 10^7^ bacteria per gram of root and that a mutant defective in *pcpI* was equally competitive than the wild strain in the colonization of the total root and root tips (Supp. Fig. S11). Subsequently, we evaluated *in vitro* whether maize root exudates serve as attractants for *P*. *putida* 1290. Quantitative capillary assays revealed that root exudates strongly attracted *P*. *putida* 1290 and that the magnitude of this attraction increased with the concentration of root exudates (Supp. Fig. S12). However, the *pcpI* mutant and the wild type strain exhibited similar chemotaxis to maize root exudates (Supp. Fig. S12).

## Discussion

IAA is one of the central signal molecules of life. This auxin is synthesized in all kingdoms of life (Oliveira *et al*., 2007; Aklujkar *et al*., 2014; Bogaert *et al*., 2019; Duca and Glick, 2020; Gallei *et al*., 2020) and exerts a variety of different biological functions, including the regulation of (i) inflammatory responses in humans (Addi *et al*., 2019): (ii) growth and development in plants (Zhao, 2018; Gallei *et al*., 2020) and algae (Ohtaka *et al*., 2017; Bogaert *et al*., 2019); (iii) hyphal growth and sporulation in fungi (Fu *et al*., 2015; Nicastro *et al*., 2021); and (iv) bacterial physiology and metabolism (Duca and Glick, 2020). Notably, the role of IAA as an intra‐ and inter‐kingdom signal molecule has been investigated primarily in model systems based on bacteria–plant interactions, where it has been shown to act as a key signal in the modulation of various phytostimulatory and phytopathogenic processes through various mechanisms that include the alteration of auxin homeostasis and disturbances of auxin signalling in their plant hosts (Spaepen and Vanderleyden, 2011; Duca *et al*., 2014; Kunkel and Harper, 2018; Duca and Glick, 2020).

We identify here the first bacterial IAA chemoreceptor; a finding that expands the range of chemoreceptors that recognize central signal molecules of life, such as receptors for histamine (Corral‐Lugo *et al*., 2018), putrescine (Corral‐Lugo *et al*., 2016) or γ‐aminobutyrate (Rico‐Jimenez *et al*., 2013). Importantly, PcpI recognized and mediated chemoattraction to another important signal molecule, salicylate. Salicylate is an essential phytohormone that promotes plant immune responses against pathogens, as well as regulates plant growth, flowering and senescence (Bakker *et al*., 2014; Peng *et al*., 2021). Salicylate production has been described in bacteria and fungi (Bakker *et al*., 2014; Mishra and Baek, 2021) and its biosynthesis in bacteria is mainly associated with the production of salicylate‐based siderophores (Miethke and Marahiel, 2007; Bakker *et al*., 2014). However, current data support the role of salicylate as a central bacterial signal molecule, since it was shown to regulate antibiotic resistance, secondary metabolism, biofilm formation and virulence, among other processes (Price *et al*., 2000; Bakker *et al*., 2014; Lowe‐Power *et al*., 2016; Matilla *et al*., 2022). Notably, we have published recently a catalogue of signal molecules that are recognized by bacterial chemoreceptors, sensor kinases and transcriptional regulators, and salicylate was among the signal molecules for which the highest number of different sensor domains has been identified, namely, domains that belong to seven different Pfam families (Matilla *et al*., 2022). The PcpI LBD is un‐annotated in Pfam, suggesting that the diversity of salicylate binding domains can be even larger. Although PcpI‐LBD recognized salicylate with a modest affinity (*K*_D_ = 826 ± 34 μM), the onset of chemotactic responses occurred at much lower concentrations, namely, 10 μM (Fig. 6). These discrepancies may be due to signal amplification in chemosensory arrays observed previously in *Escherichia coli* (Sourjik and Berg, 2002), the model bacterium for studying chemotaxis signal transduction (Parkinson *et al*., 2015). Salicylate can be detected in plant fluids and tissues at concentrations of up to 600 μM (Smith‐Becker *et al*., 1998; Huang *et al*., 2006; Ratzinger *et al*., 2009), indicating that PcpI mediates chemotaxis to physiological concentrations of this plant hormone.

Chemotaxis towards different phytohormones, including salicylate (Fernandez *et al*., 2017), ethylene (Kim *et al*., 2007) and jasmonic acid (Antunez‐Lamas *et al*., 2009) has been described in several plant‐associated bacteria, and the corresponding chemoreceptors involved identified (Kim *et al*., 2007; Rio‐Alvarez *et al*., 2015; Fernandez *et al*., 2017). However, to the best of our knowledge, PcpI is the first chemoreceptor that mediates chemotaxis towards two different phytohormones. The mechanisms by which IAA is sensed by bacteria remain mostly unknown. In *E*. *coli*, the tryptophan repressor TrpR recognizes IAA with low affinity (Marmorstein *et al*., 1987) and antibiotic synthesis in *Serratia plymuthica* is controlled by the transcriptional regulator AdmX, which binds IAA with significant affinity (*K*_D_ = 15.2 μM) (Matilla *et al*., 2018). Our data strongly indicate that IAA and salicylic acid employ two different mechanisms to activate PcpI. Whereas salicylate activates PcpI by binding to the LBD, the mode of receptor stimulation by IAA is different since it does not involve direct recognition by the LBD (Fig. 5). Chemotaxis towards the hormone norepinephrine in *E*. *coli* was found to require its metabolization to 3,4‐dihydroxymandelic acid – a metabolite that was proposed to be the chemoeffector recognized by the Tsr chemoreceptor (Pasupuleti *et al*., 2014). However, the fact that mutation of the *iac* gene cluster does not affect the chemotactic properties of 1290 towards IAA, as well as the finding that *in trans* expression of *pcpI* in KT2440 and PAO1 conferred IAA chemotaxis to both strains strongly indicates that this tactic behaviour is not dependent on the sensing of an IAA catabolic intermediate.

For the large majority of the characterized chemoreceptors a single mode of activation, namely, by signal binding to the receptor LBD, has been reported (Ortega *et al*., 2017; Matilla *et al*., 2021a). However, studies of the two primary chemoreceptor models, *E*. *coli* Tar and Tsr, has revealed that both receptors can be activated by the direct binding of l‐Asp and l‐Ser, as well as by the recognition of the SBPs MBP and LsrB in complex with maltose and autoinducer‐2, respectively (Zhang *et al*., 1999; Hegde *et al*., 2011; Laganenka *et al*., 2016). Further research is necessary to identify the mode of PcpI activation by IAA, but current data indicate a convergent evolution of two different mechanisms that permit the sensing of two phytohormones. In accordance, an IAA binding SBP, Dde_0634, has been identified in an environmental isolate of *Desulfovibrio desulfuricans* (Vetting *et al*., 2015) and the SBP IaaM from the IAA‐degrading bacterium *Azoarcus evansii* was predicted to be involved in the uptake of IAA (Ebenau‐Jehle *et al*., 2012). However, the analysis of the genome of *P*. *putida* 1290 did not reveal the presence of any SBP homologous to Dde_0634 or IaaM, making targeted analysis of any candidate IAA binding SBPs unfeasible. SBP expression is tightly regulated (Matilla *et al*., 2021b) and the failure of our pull‐down experiments to detect an SBP that interacts with PcpI may be due to a very low cellular abundance. SBP‐mediated receptor stimulation has been proposed to expand the diversity of chemoeffectors recognized by chemoreceptors as well as their ligand concentration range (Matilla *et al*., 2021b). IAA can be found in plant cells, organic soils and in the rhizosphere at concentrations in the micromolar range (Brandl and Lindow, 1998; Petersson *et al*., 2009; Greenhut *et al*., 2018); values that are in the same range as the IAA concentrations for which taxis was observed (Figs. 1 and 3).

Current data support that chemotaxis represents an evolutionary advantage for bacteria that establish interactions with plants, being essential for plant colonization and infection in several bacterial species (Corral‐Lugo *et al*., 2016; Matilla and Krell, 2018; Compton and Scharf, 2021; Sanchis‐López *et al*., 2021). Indeed, 81% of the plant‐associated bacteria have chemoreceptor genes, which are superior to the bacterial average of 47% (Sanchis‐López *et al*., 2021). Furthermore, phytobacteria possess twice as many chemoreceptors than bacteria classified as non‐plant‐associated (Sanchis‐López *et al*., 2021). This prevalence of chemoreceptor genes in phytobacteria may be linked to the physical and chemical complexity of the plant environment as well as to the high competitiveness that exists in plant‐associated niches such as the rhizosphere (Raina *et al*., 2019; Fitzpatrick *et al*., 2020; Sanchis‐López *et al*., 2021). In this regard, a growing body of data reveals the importance of chemotaxis towards specific nutrients for an efficient plant colonization by beneficial and pathogenic phytobacteria. In this chemotaxis‐mediated host colonization, amino acids, organic acids and sugars were found to play major roles (Oku *et al*., 2012, 2014; Hida *et al*., ; Cerna‐Vargas *et al*., 2019; Feng *et al*., 2019; O'Neal *et al*., 2020; Compton and Scharf, 2021). However, determining the role of chemotaxis towards alternative plant molecules (e.g. fatty acids, nucleotides, host hormones, inorganic nutrients) and the biological function of specific chemoreceptors remains challenging. For example, chemotaxis to root exudates required multiple chemoreceptors in *Bacillus subtilis*, namely McpB, McpC and TlpC. In contrast, a triple deletion mutant defective in these chemoreceptors colonized plant roots at the wild type levels (Allard‐Massicotte *et al*., 2016). Root colonization is a multifactorial process (Jones *et al*., 2019; Knights *et al*., 2021) and current research supports that the combined action of chemoreceptors with complementary functions is responsible for chemotaxis towards roots as a prior step for plant colonization (Allard‐Massicotte *et al*., 2016; Feng *et al*., 2019). In this context, under the experimental conditions tested, PcpI did not play a relevant role in plant root colonization (Suppl. Fig. S11). This aspect may be associated with the remarkable number and diversity of chemoreceptors encoded in the genome of *P*. *putida* 1290 and the chemical composition of maize root exudates; which major constituents are sugars, amino and organic acids (Fan *et al*., 2012; da Silva Lima *et al*., 2014; Lopez‐Farfan *et al*., 2019). However, the composition of plant exudates varies qualitatively and quantitatively according to physical, chemical and biological factors (Sasse *et al*., 2018; Vives‐Peris *et al*., 2020; Compton and Scharf, 2021). Alterations in metabolite exudation influences plant microbiome composition (Sasse *et al*., 2018; Pascale *et al*., 2020) and chemotactic recruitment of bacteria is dependent on variations in the composition of plant exudates (Feng *et al*., 2019; Compton and Scharf, 2021). It can therefore be hypothesized that PcpI may play a role under plant‐specific physiological conditions, for example, during the induction of systemic acquired resistance when strong increases in salicylic acid levels have been measured in plant fluids (Smith‐Becker *et al*., 1998).

Salicylate and IAA served as nutrient source for *P*. *putida* 1290 (Supp. Fig. S1) and migration mediated by chemotaxis or energy taxis towards these compounds may confer a selective advantage over microbial competitors in specific niches with significant concentrations of these PcpI ligands. In accordance, bacterial IAA metabolism was demonstrated to act as a metabolic signal interference altering the communication networks between competitor bacteria and their plant hosts (Finkel *et al*., 2020). The wide distribution of IAA catabolic genes in bacteria (Li *et al*., 2016; Laird *et al*., 2020) has raised questions about their ecological role and further research will establish whether chemotaxis to IAA is a general feature of IAA degrading bacteria.

## Experimental procedures

### Bacterial strains, plasmids and culture conditions

Bacterial strains and plasmids are listed in Supp. Table S2. *Pseudomonas putida* and *P*. *aeruginosa* strains were grown routinely at 30°C and 37°C, respectively, in LB or M9 minimal medium supplemented with 1 mM MgSO_4_, 6 mg L^−1^ Fe‐citrate, 15 mM glucose as carbon source and trace elements as described previously (Abril *et al*., 1989). *Escherichia coli* strains were grown at 37°C. *Escherichia coli* DH5α was used as a host for gene cloning. Media for propagation of *E*. *coli* β2163 were supplemented with 300 μM 2,6‐diaminopimelic acid. When necessary, antibiotics were used at the following final concentrations: kanamycin, 50 μg ml^−1^, ampicillin, 100 μg ml^−1^, gentamycin 10 μg ml^−1^ (*E*. *coli*) or 100 μg ml^−1^ (*P*. *putida* and *P*. *aeruginosa*), streptomycin, 50 μg ml^−1^. Sucrose was added to a final concentration of 10% (wt/vol) when required to select derivatives that had undergone a second crossover event during marker‐exchange mutagenesis.

### Construction of bacterial strains and complementation plasmid

Mutants defective in *iacA*, *E6B08_RS07220*, *E6B08_RS17840*, *E6B08_RS22475*, *E6B08_RS29420* and *E6B08_RS30830* were constructed using derivate plasmids of pCHESIΩKmGm. These plasmids are listed in Supp. Table S2 and were generated by amplifying a 0.6–0.9 kb region of the gene to be mutated using primers listed in Supp. Table S3. The PCR products were then cloned into pCHESIΩKmGm in the same transcriptional direction as the P_*lac*_ promoter using the enzymes specified in Supp. Table S2. A plasmid‐free mutant defective in *pcpI* was constructed by homologous recombination using a derivative plasmid of the suicide vector pKNG101. The plasmid for the construction of this *pcpI* deletion mutant was generated by amplifying the up‐ and downstream flanking regions of the *pcpI* gene using the primers listed in Supp. Table S3. The resulting PCR products were digested with the enzymes specified in Supp. Table S2 and ligated in a three‐way ligation into pUC18Not, previously cloned into the marker exchange vector pKNG101. In all cases, plasmids for mutagenesis were transferred to *P*. *putida* strains by biparental conjugation using *E*. *coli* β2163. For the construction of the plasmid for complementation assays, the *pcpI* gene was amplified using primers listed in Supp. Table S3 and cloned into pBBR1MCS‐5_START to generate the plasmid pMAMV378. The resulting plasmid was transformed into *P*. *aeruginosa* and *P*. *putida* strains by electroporation. All plasmids and mutations were confirmed by PCR and sequencing.

### Swimming motility assays

*Pseudomonas putida* 1290 strains were grown overnight in M9 minimal medium containing 5 mM IAA as carbon source and adjusted to an OD_660_ of 1. Two microliters of these cultures were spotted onto minimal medium‐Difco agar [0.3% (wt/vol)] plates containing 5 mM IAA acid as sole carbon source and incubated at 30°C.

### Chemotaxis assays

Overnight cultures in M9 minimal medium were used to inoculate fresh medium to reach an OD_660_ of 0.075. Cells were cultured at 30°C (*P*. *putida*) or 37°C (*P*. *aeruginosa*) until an OD_660_ of 0.4–0.5 was reached. Subsequently, cells were washed twice by centrifugation (1667 x*g* for 5 min at room temperature) and re‐suspension in chemotaxis buffer [50 mM KH_2_PO_4_ */*K_2_HPO_4_, 20 mM EDTA, 0.05% (vol/vol) glycerol, pH 7.0], and then re‐suspended in the same buffer to reach an OD_660_ of 0.1. Aliquots (230 μl) of the resulting cell suspension were placed into the wells of a 96‐well microtiter plate. One microliter capillaries (Microcaps, Drummond Scientific, Ref. P1424) were heat‐sealed at one end and filled with buffer (control) or chemoeffector solutions prepared in chemotaxis buffer. The capillaries were rinsed with sterile water and immersed into the bacterial suspensions at its open end. After 30 min, capillaries were removed from the wells, rinsed with sterile water, and emptied into 1 ml of chemotaxis buffer. Serial dilutions were plated onto M9 minimal medium plates supplemented with 15 mM glucose and incubated at 30°C or 37°C. Colony forming units (CFU) counts were determined and corrected with the number of cells that swam into buffer containing capillaries. Data are means and standard deviations of three biological replicates conducted in triplicate.

### RNA extraction, cDNA synthesis and quantitative real‐time PCR analyses

RNA was extracted from mid‐logarithmic growth phase cultures grown in minimal medium by the hot phenol method using the TRI® Reagent protocol (Ambion) according to the manufacturer's instructions. RNA concentration was determined spectrophotometrically using a NanoDrop spectrophotometer (Thermo Scientific) and RNA integrity was assessed by agarose gel electrophoresis. Genomic DNA contamination was eliminated by treating total RNA with Turbo DNA‐free (Ambion), followed by a purification with RNeasy mini kit (Qiagen). The synthesis of cDNA was performed using 200 ng of random hexamer primers (Roche) and SuperScript II reverse transcriptase (Invitrogen) in a 20 μl reaction with 1 μg of total RNA and incubation at 42°C for 1.5 h. Quantitative real‐time PCR amplifications were performed using the iQ™ SYBR® Green supermix (Bio‐Rad) in a MyiQ2 system (Bio‐Rad) associated with iQ5 optical system software (version 2.1.97.1001). PCR reactions contained 6.25 μl of 2× SYBR Green supermix, 400 nM of each primer and 0.5 μl of cDNA in a final volume of 12.5 μl. The PCR protocol used was as follows: one cycle at 95°C for 5 min followed by 40 cycles at 95°C for 15 s, 63°C for 30 s, and 72°C for 20 s and melting curve analysis from 55°C to 95°C, with an increment of 0.5°C/10 s for 80 cycles. The primers used in this study were designed using the Clone Manager software 6.0 (Sci‐Ed Software) and are listed in Supp. Table S3. Standard curves for each primer pair were generated with serial dilutions of genomic DNA to determine PCR efficiency and melting curve analyses were conducted to ensure amplification of a single product. The relative gene expression was calculated using the critical threshold (∆Ct) method (Silver *et al*., 2006) using *gyrB* as the internal control to normalize the data. Data are the means and standard deviations of three biological replicates conducted in triplicate.

### Construction of overexpression plasmids, protein expression and purification

The DNA fragments encoding the LBD of the chemoreceptor PcpI (amino acids 38–174) and the SBP E6B08_RS28125 were amplified by PCR from genomic DNA and primers listed in Supp. Table S3. The PCR products were then cloned into the NdeI and BamHI sites of pET28b(+) to generate plasmids pMAMV365 and pMAMV385, respectively. The sequence predicted to be signal peptide was not included in pMAMV385. *Escherichia coli* BL21 (DE3) harbouring plasmids pMAMV365 and pMAMV385 were grown under continuous shaking (200 rpm) at 30°C in 2 L Erlenmeyer flasks containing 500 ml LB medium supplemented with kanamycin. At an OD_660_ of 0.6, PcpI‐LBD and E6B08_RS28125 expression was induced by the addition of 0.25 mM isopropyl ß‐D‐1‐thiogalactopyranoside (IPTG). Growth was continued at 18°C overnight and cells were harvested by centrifugation at 10 000 x*g* for 20 min at 4°C. Proteins were purified by metal affinity chromatography using standard procedures. Briefly, cell pellets for the purification of PcpI‐LBD and E6B08_RS28125 were re‐suspended in buffer A [20 mM Tris, 500 mM NaCl, 10 mM imidazole, 1 mM EDTA, 5% (vol/vol) glycerol, pH 8.0] and buffer B [50 mM Tris, 150 mM NaCl, 10 mM imidazole, 10% (vol/vol) glycerol, pH 8.0] respectively, containing cOmplete™ protease inhibitor cocktail (Roche) and benzonase (Sigma‐Aldrich). Cells were broken by French press treatment at a gauge pressure of 62.5 lb in^−2^. After centrifugation at 10 000 x*g* for 1 h, the supernatants were loaded onto a 5‐ml HisTrap column (Amersham Bioscience) equilibrated with the corresponding buffers A and B, and proteins were eluted by a linear gradient of 40–500 mM imidazole in the same buffers.

### Differential scanning fluorimetry‐based thermal shift assays

Using DSF, changes in the midpoint of protein unfolding transition (Tm) of a protein can be recorded. Typically, ligand binding stabilizes the protein and the identification of compounds that cause an increase in the Tm value is an evidence for ligand binding (Martin‐Mora *et al*., 2018a). DSF assays were performed using a Bio‐Rad MyiQ2 Real‐Time PCR instrument. Ligands from different compound arrays (Biolog, Hayward, CA, USA; for further information, refer to http://www.biolog.com/) were dissolved in 50 μl of Milli‐Q water, which, according to the manufacturer, corresponds to a concentration of 10–20 mM. Assay mixtures (25 μl) contained 20–50 μM protein dialyzed in buffer C [50 mM Tris, 150 mM NaCl, 5% (vol/vol) glycerol, pH 8.0; PcpI‐LBD] or buffer D [5 mM Tris, 5 mM Pipes, 5 mM Mes, 10% glycerol (vol/vol), 150 mM NaCl, pH 8; E6B08_RS28125], SYPRO® Orange (Life Technologies) at 5× concentration and ligands at final concentrations of 1–2 mM. Samples were heated from 23°C to 85°C at a rate of 1°C min^−1^. The protein unfolding curves were obtained by monitoring the changes in SYPRO Orange fluorescence. Tm values correspond to the minima of the first derivatives of the raw fluorescence data.

### Isothermal titration calorimetry

Measurements were made using a VP‐ITC titration calorimeter (Microcal, Northampton, MA, USA) at a temperature of 25°C. PcpI‐LBD and E6B08_RS28125 were dialyzed into buffer C and buffer D, respectively, and proteins at 40–226 μM were placed into the sample cell and titrated with 3.2–9.6 μl aliquots of 0.5–5 mM ligand solutions freshly made up in dialysis buffer. In the absence of binding, the experiment was repeated at an analysis temperature of 10°C. The mean enthalpies measured from the injection of effectors into the buffer were subtracted from raw titration data prior to data analysis with the MicroCal version of ORIGIN. Data were fitted with the ‘One binding site model’ of ORIGIN.

### Pull‐down assays

Overnight cultures of *P*. *putida* 1290 grown in M9 minimal medium supplemented with glucose as a carbon source was used to inoculate fresh medium to reach an OD_660_ of 0.075. After overnight growth, cultures were diluted to an OD_600_ of 0.075 in the same medium until an OD_660_ of 0.6. Subsequently, pellets were re‐suspended in buffer A containing 10 mM IAA and broken by French press treatment at a gauge pressure of 62.5 lb in^−2^. After centrifugation at 10 000 x*g* for 1 h, the supernatant was loaded onto a HisTrap column on which PcpI‐LBD had previously been immobilized. The column was washed with buffer A prior to protein elution using a 0–6 M guanidine hydrochloride gradient in buffer A. Finally, to release PcpI‐LBD or any other protein bound to the HisTrap column, a gradient of 10–500 mM imidazole in buffer A was applied. As a control, the *P*. *putida* 1290 supernatant was applied to a column that did not contain PcpI‐LBD. Bands of interest were excised from an SDS‐PAGE gel, digested with trypsin and analyzed by MALDI‐TOF mass spectrometry at the proteomics service of the Faculty of Pharmacy – Complutense University of Madrid (Spain). Protein identity was established using the MASCOT software.

### Competitive root colonization assays

Maize seeds were sterilized and germinated as described previously (Matilla *et al*., 2007). Thereafter, germinated seeds were planted at the centre of a 50 ml Sterilin tubes containing 40 g of sterile washed silica sand. For the competitive root colonization assays, 100 μl of a 10^7^ CFU ml^−1^ 1:1 mixture of the wild type *P*. *putida* 1290 and a *pcpI* mutant were inoculated at the edge of each Sterilin tube. Subsequently, plants were maintained at 24°C with a daily light period of 16 h. After 10 days, bacterial cells were recovered from the rhizosphere or from 1 mm of the main root apex, as described previously (Matilla *et al*., 2007). Serial dilutions were plated in minimal medium‐agar and minimal medium‐agar supplemented with 50 μg ml^−1^ of kanamycin to select the *pcpI* mutant strain.

### Collection of maize root exudates

The collection of maize root exudates was carried out as previously indicated (Lopez‐Farfan *et al*., 2019). Briefly, maize seeds were sterilized and germinated as described previously (Matilla *et al*., 2007). Sixteen germinated seeds were transferred into an axenic system with 450 ml of sterile water and allowed to grow at room temperature. After 8 days, the water containing root exudates was collected and vacuum filtrated (0.45 μm cut‐off). An aliquot was taken and plated onto solid LB media to check for contamination. Maize root exudates were aliquoted, freeze‐dried and stored at −80°C. Before use, the lyophilized exudates were re‐suspended in chemotaxis medium and filter‐sterilized.

### Growth experiments

*Pseudomonas putida* 1290 strains were grown overnight in an M9 minimal medium containing 15 mM glucose. Cultures were washed twice with M9 salts medium and then diluted to an OD_600_ of 0.02 in M9 containing 5 mM glucose (positive control) and medium supplemented with 5 mM IAA, benzoate, 3‐MBA and salicylate as carbon sources. Two‐hundred microliters of these cultures were transferred to microwell plates and growth (OD_600_) at 30°C was followed over time using Bioscreen Microbiological Growth Analyser (Oy Growth Curves Ab, Helsinki, Finland).

## Supporting information


**Appendix S1**: Supporting Information.
