# Supplementary material for: A bacterial chemoreceptor that mediates chemotaxis to two different plant hormones
Source: Environ Microbiol. 2022 Feb 1;24(8):3580–97. doi: 10.1111/1462-2920.15920 (PMC9543091; doi:10.1111/1462-2920.15920)
Supplement: Supplementary file 1 — Appendix S1: Supporting Information. [file EMI-24-3580-s001.pdf]

Supplementary material

to

A bacterial chemoreceptor that mediates chemotaxis to two different plant hormones

Miriam Rico-Jiménez[a], Amalia Roca[b], Tino Krell[a], Miguel A. Matilla[a#]

[a]Department of Environmental Protection, Estación Experimental del Zaidín, Consejo Superior de Investigaciones Científicas, Granada, Spain.

[b]Department of Microbiology, Facultad de Farmacia, Campus Universitario de Cartuja, Universidad de Granada, 18071 Granada, Spain.

Running Head: Chemotaxis to plant hormones

[#]Address correspondence to Miguel A. Matilla, miguel.matilla@eez.csic.es

Supplementary Table S1. Thermodynamic parameters derived from the microcalorimetric titrations of PcpI-LBD and E6B08_RS28125 with different ligands. Data were analyzed using the "One binding site model" of the MicroCal version of ORIGIN. The corresponding data are shown in Figures 5 and supplementary Figure S6.

Protein/Ligand	K_D (µM)	ΔH (kcal/mol)
**PcpI-LBD**[a]		
Salicylate	826 ± 34	-14.5 ± 5.7
Benzoate	171 ± 14	-0.75 ± 0.03
3-Methylbenzoate	91 ± 8	-0.37 ± 0.02
**E6B08_RS28125**[b]		
L-ornithine	0.93 ± 0.11	-10.13 ± 0.42
L-histidine	3.28 ± 0.29	-8.43 ± 0.72
L-arginine	29.5 ± 2.61	8.61 ± 0.3

[a]No binding was detected for 2-aminobenzoate, 2-chlorobenzoate, 2-nitrobenzoate, 3-aminobenzoate, 3-nitrobenzoate, 4-hidroxybenzoate, acetyl salicylic acid, benzene, protocatechuate, vanillate, vanillin and quinate.
[b]No binding was detected for L-Lysine.

Supplementary Table S2. Strains and plasmids used in this study.

Strains and plasmids	Genotype or relevant characteristics[a]	Ref.
**Strains**		
Escherichia coli BL21(DE3)	F$^-$ *ompT gal dcm lon hsdS*$_B$($r_B^- m_B^-$) λ(DE3 [*lacI lacUV5-T7p07 ind1 sam7 nin5*]) [*malB*$^+$]$_{K-12}$(λ^S)	(Jeong *et al.*, 2009)
E. coli DH5α	F$^-$ *endA1 glnV44 thi-1 recA1 relA1 gyrA96 deoR nupG purB20* φ80d*lacZ*ΔM15 Δ(*lacZYA-argF*)U169, hsdR17($r_K^- m_K^+$), λ$^-$	(Woodcock *et al.*, 1989)
E. coli CC118λ*pir*	*araD* Δ(*ara, leu*) Δ*lacZ74 phoA20 galK thi-1 rspE rpoB argE recA1* λ*pir*	(Herrero *et al.*, 1990)
E. coli β2163	F- RP4-2-Tc::Mu Δ*dapA*::(*erm-pir*), Km^R, Em^R	(Demarre *et al.*, 2005)
Pseudomonas putida 1290	Wild type strain	(Laird and Leveau, 2019)
P. putida 1290-CheA	*cheA*::pCHESIΩKmGm; Km^R, Gm^R	This study
P. putida 1290-IacA	*iacA*::pCHESIΩKmGm; Km^R, Gm^R	This study
P. putida 1290-RS07220	*E6B08_RS07220*::pCHESIΩKmGm; Km^R, Gm^R	This study
P. putida 1290-RS17840	*E6B08_RS17840*::pCHESIΩKmGm; Km^R, Gm^R	This study
P. putida 1290-RS22475	*E6B08_RS22475*::pCHESIΩKmGm; Km^R, Gm^R	This study
P. putida 1290-PcpI	Δ*pcpI*::Km; Km^R	This study
P. putida 1290-RS29420	*E6B08_RS29420*::pCHESIΩKmGm; Km^R, Gm^R	This study
P. putida 1290-RS30830	*E6B08_RS30830*::pCHESIΩKmGm; Km^R, Gm^R	This study
Pseudomonas aeruginosa PAO1	Wild type strain	(Stover *et al.*, 2000)
Pseudomonas putida KT2440	Wild-type, non-pathogenic soil bacterium	(Belda *et al.*, 2016)
**Plasmids**		
pET28b(+)	Km^R; Protein expression plasmid	Novagen
pMAMV365	Km^R; pET28b(+) derivative containing a DNA fragment encoding PcpI-LBD cloned into the NdeI/BamHI	This study
pMAMV385	Km^R; pET28b(+) derivative containing a DNA fragment encoding *E6B08_RS28125* cloned into the NdeI/BamHI sites. The sequence predicted to be signal peptide was not included.	This study
pBBR1MCS-5_START	Gm^R; *oriRK2 mobRK2*	(Obranic *et al.*, 2013)
pCHESIΩGmKm	Ap^R, Km^R, Gm^R; pUNφ18 bearing a HindIII fragment from pHP45ΩKm (Ω-KmGm interposon) cloned at the HindIII site, *oriT* RP4	P. van Dillewijn, unpublished data
pMAMV352	Km^R, Gm^R; PCR product containing a 0.8 kb region of *cheA* was inserted into the EcoRI/SacI sites of pCHESIΩKmGm	This study
pMAMV353	Km^R, Gm^R; PCR product containing a 0.8 kb region of *iacA* was inserted into the EcoRI/SacI sites of pCHESIΩKmGm	This study
pMAMV355	Km^R, Gm^R; PCR product containing a 0.9 kb region of *E6B08_RS30830* was inserted into the EcoRI/SacI sites of pCHESIΩKmGm	This study
pMAMV356	Km^R, Gm^R; PCR product containing a 0.7 kb region of *E6B08_RS17840* was inserted into the EcoRI/SacI sites of pCHESIΩKmGm	This study
pMAMV357	Km^R, Gm^R; PCR product containing a 0.7 kb region of *E6B08_RS22475* was inserted into the EcoRI/SacI sites of pCHESIΩKmGm	This study
pMAMV358	Km^R, Gm^R; PCR product containing a 0.7 kb region of *E6B08_RS29420* was inserted into the EcoRI/SacI sites of pCHESIΩKmGm	This study

pMAMV361	Km^R, Gm^R; PCR product containing a 0.7 kb region of *E6B08_RS07220* was inserted into the EcoRI/SacI sites of pCHESIΩKmGm	This study
pUC18Not	Ap^R; identical to pUC18 but with two NotI sites flanking pUC18 polylinker	(Herrero *et al.*, 1990)
p34S-Km3	Km^R, Ap^R; *Km3* antibiotic cassette	(Dennis and Zylstra, 1998)
pMAMV366	Ap^R; 1.5-kb PCR product containing a 1086 bp deletion of *pcpI* inserted into the EcoRI/HindIII sites of pUC18Not	This study
pMAMV367	Ap^R, Km^R; 0.95-kb BamHI fragment containing *km3* cassette of p34S-Km3 was inserted into BamHI site of *pcpI* in pMAMV366	This study
pKNG101	Sm^R; *oriR6K mob sacBR*	(Kaniga *et al.*, 1991)
pMAMV368	Sm^R, Km^R; 2.5-kb NotI fragment of pMAMV367 was cloned at the same site in pKNG101	This study
pMAMV378	Gm^R; a 1.7-kb PCR fragment containing the *pcpI* gene cloned into the NdeI/BamHI sites of pBBR1-MCS-5_START	This study

[a]Ap, ampicillin; Km, kanamycin; Gm, gentamycin; Sm, streptomycin; Em, erythromycin.

Supplementary Table S3. Oligonucleotides used in this study.

Oligonucleotide	Sequence (5'-3')	Purpose
cheA-1290-EcoRI-F	TAATGAATTCCTTCAGCTCAACGAGCTGGTGG	Construction of *cheA* mutant
cheA-1290-SacI-R	TAATGAGCTCGTGCTCGCTGATGTCGTCACC	
iacA-1290-EcoRI-F	TAATGAATTCCGCTTCGGTGGACTGGAGTG	Construction of *iacA* mutant
iacA-1290-SacI-R	TAATGAGCTCCAGGTTGGTGACGTCGACCG	
MCP30830-EcoRI-F	TAATGAATTCGAGGCGCTACTGGCCAACG	Construction of *E6B08_RS30830* mutant
MCP30830-SacI-R	TAATGAGCTCACATCTCGTGCAAGGCCGTG	
MCP17840-EcoRI-F	TAATGAATTCCCGCAACCTGGACATGCTCA	Construction of *E6B08_RS17840* mutant
MCP17840-SacI-R	TAATGAGCTCGGTGCTCACCGCGTTGTTGG	
MCP22475-EcoRI-F	TAATGAATTCCGGCCAACGTCGACACCTATG	Construction of *E6B08_RS22475* mutant
MCP22475-SacI-R	TAATGAGCTCGCATTGCGCGCCACTTCATC	
MCP29420-EcoRI-F	GTCGACGAATTCCAGCAGATGAC	Construction of *E6B08_RS29420* mutant
MCP29420-SacI-R	TAATGAGCTCCTCGTCGTGGCTGGTCACC	
MCP07220-EcoRI-F	TAATGAATTCGCGAGAACCTGATCCTG	Construction of *E6B08_RS07220* mutant
MCP07220-SacI-R	TAATGAGCTCGACCTTCTGGTTGTTGGC	
RS28110-EcoRI-F	TAATGAATTCGAAAGCGTGCGCGAGAACC	Construction of *E6B08_RS28110* (*pcpI*) mutant
RS28110-BamHI-R	TAATGGATCCCATGCGCTGGATCTGCTGC	
RS28110-BamHI-F	TAATGGATCCGCGATCCAGGCAATGAACCAG	
RS28110-HindIII-R	TAATAAGCTTCCGACATCAACGCCTACCGC	
RS28110-NdeI-F	TAATCATATGCGGGGCATGGACGGCATC	Construction of pET28-PcpI-LBD (pMAMV365)
RS28110-BamHI-R	TAATGGATCCTCAGGTCTTGGCCTGGCGCGCATC	
RS28110-comple-NdeI-F	TAATCATATGTACCGATGGTTTGCCCAATC	Construction of pBB1-MCS5_START-pcpI (pMAMV378) for complementation assays
RS28110-Comple-BamHI-R	TAATGGATCCGATCACGGCAAGCTGCTCG	
E6B08_RS28125-NdeI-F	AAACATATGATCGACGATGCGGTCAAGC	Construction of pET28-E6B08_RS28125 (pMAMV385)
E6B08_RS28125-BamHI-R	AAAGGATCCTTATTCCATTTCCTTCAGCCAG	
E6B08_05770-qPCR-F	TGGCCTGGTGCAGATCATCA	qPCR analyses
E6B08_05770-qPCR-R	AACAGGTCCGACAGCGACTT	qPCR analyses
E6B08_23075-qPCR-F	GACAGCCTTGGCGATAACGG	qPCR analyses
E6B08_23075-qPCR-R	CGAGCAGTGGCAAGGCAATG	qPCR analyses
E6B08_26760-qPCR-F	CAGCCTGATCGACCGTAGCA	qPCR analyses

E6B08_26760-qPCR-R	TCGCCGTTGGCGATCATGTA	qPCR analyses
E6B08_27055-qPCR-F	TTCGCAGCAGTTCCAGAACG	qPCR analyses
E6B08_27055-qPCR-R	ACTCGGTCAGCGAAGCCTTG	qPCR analyses
gyrB-1290-qPCR-F	CGTCAGCTGCCAGAGTTGGT	qPCR analyses
gyrB-1290-qPCR-R	GGCCTCGTCGTCCTTGATGT	qPCR analyses
E6B08_13285-qPCR-F2	CTCGATGGCCAGGACCTTTC	qPCR analyses
E6B08_13285-qPCR-R2	GCTTGGGCCAACGGTAGTTC	qPCR analyses
E6B08_28110-qPCR-F2	CGACAGCTCTTCGCCGACTA	qPCR analyses
E6B08_28110-qPCR-R2	GCGGACCTTGGTCTGGGAAAC	qPCR analyses

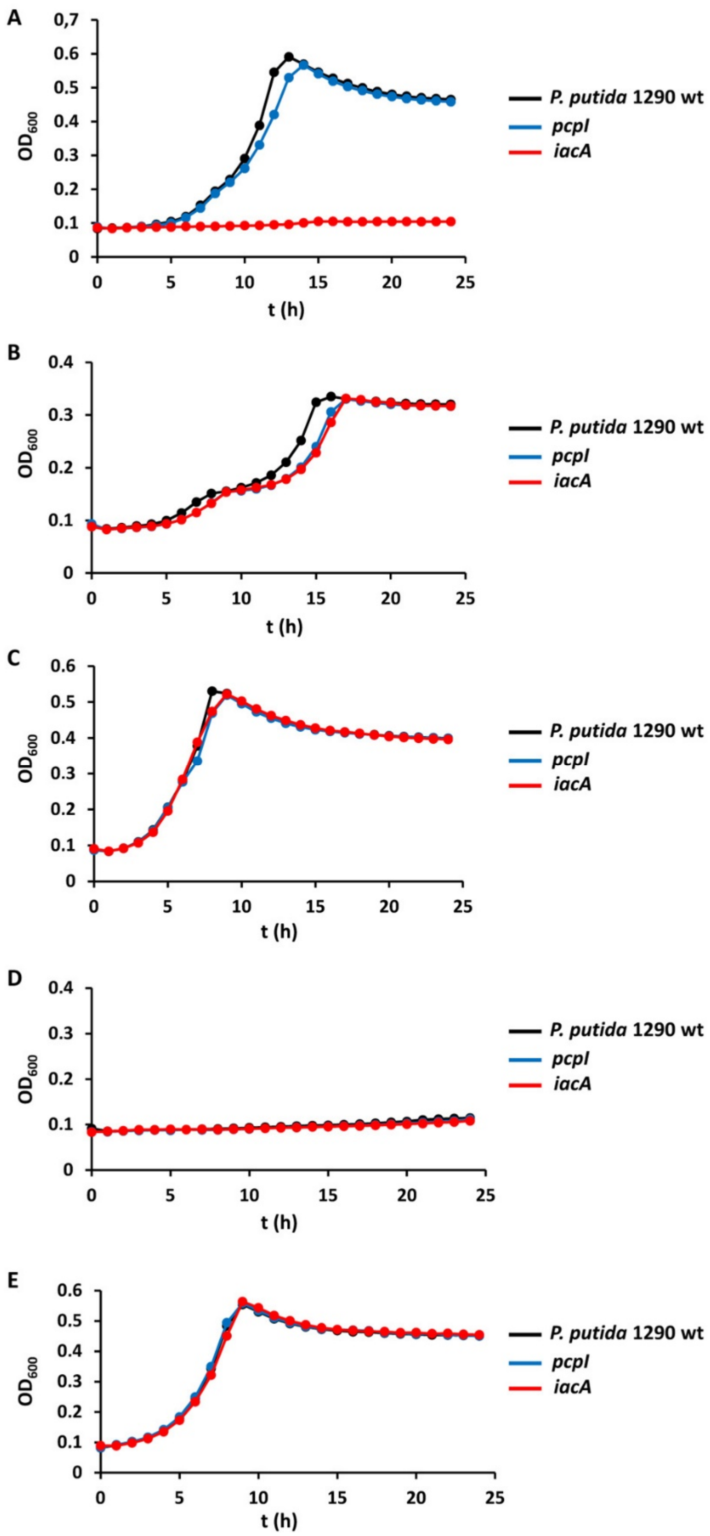

Supplementary Figure S1. Growth of *Pseudomonas putida* 1290 strains in minimal medium supplemented with different PcpI ligands as sole carbon sources. Five millimolar of indole-3-acetic acid (A), salicylic acid (B), benzoate (C), 3-methylbenzoate (D) and glucose (E, positive control) were used. Wild type *P. putida* 1290 was grown at 30 ºC using Bioscreen Microbiological Growth Analyser (Oy Growth Curves Ab Ltd, Helsinki, Finland) under shaking. Data were registered each hour. Shown are mean and standard deviation of five biological replicates.

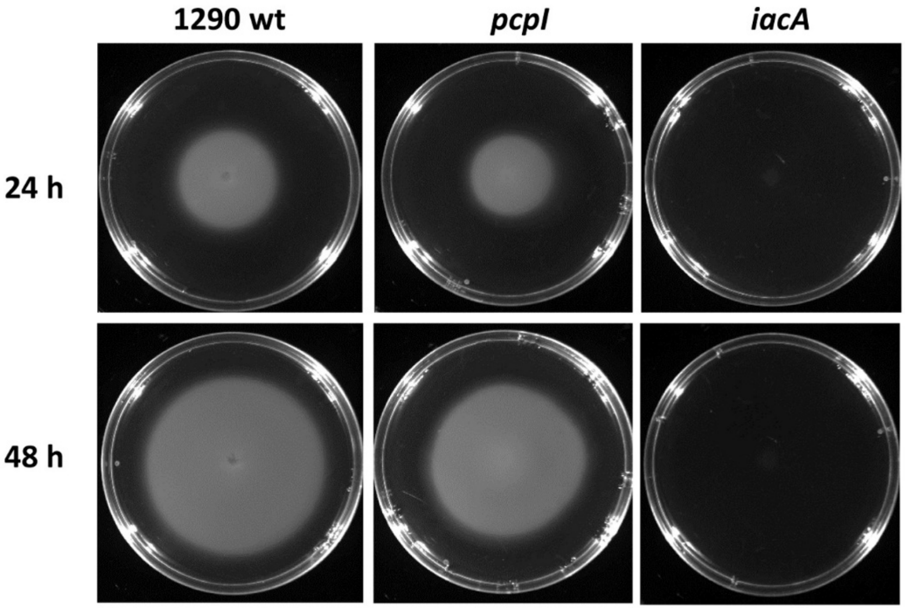

Supplementary Figure S2. Swimming plate motility assays of *Pseudomonas putida* 1290 strains grown in minimal medium supplemented with indole-3-acetic acid as sole carbon source. The bioassays were repeated at least three times, and representative results are shown.

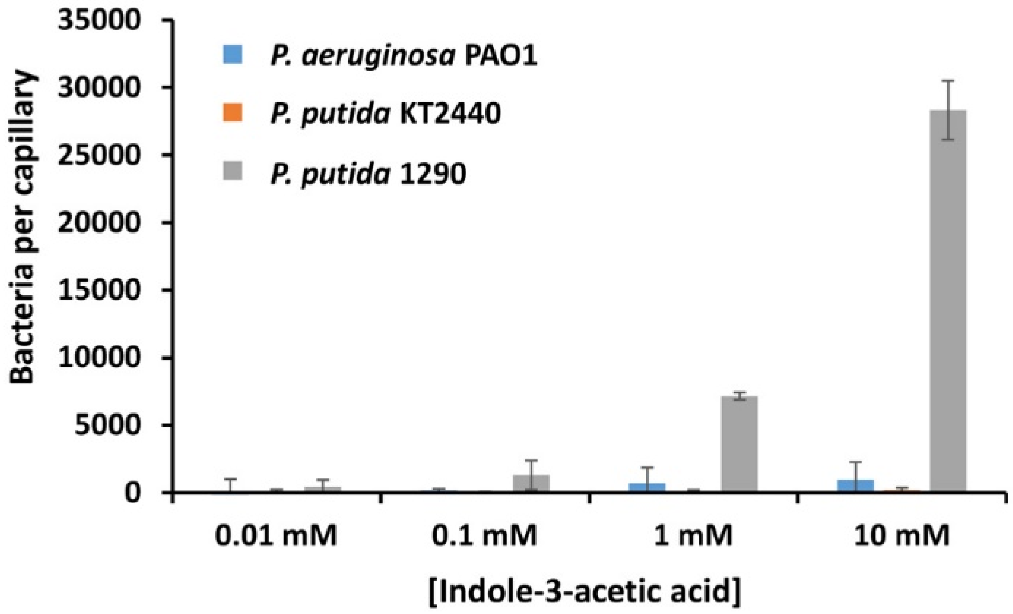

Supplementary Figure S3. Quantitative chemotaxis assays of different *Pseudomonas* strains towards different concentrations of indole-3-acetic acid. Data are means and standard deviations from three independent experiments conducted in triplicate.

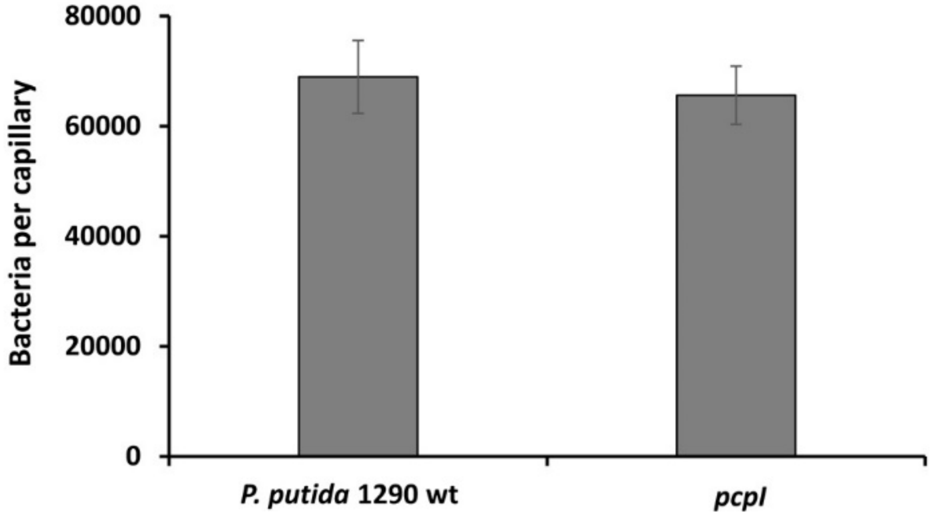

Supplementary Figure S4. The mutation of *E6B08_RS28110* (*pcpI*) has no impact on the general chemotactic responses of *P. putida* 1290. Quantitative capillary chemotaxis assays of the wild type and *pcpI* mutant strains to 0.1% (w/v) casamino acids are shown. Data are means and standard deviations from three independent experiments conducted in triplicate.

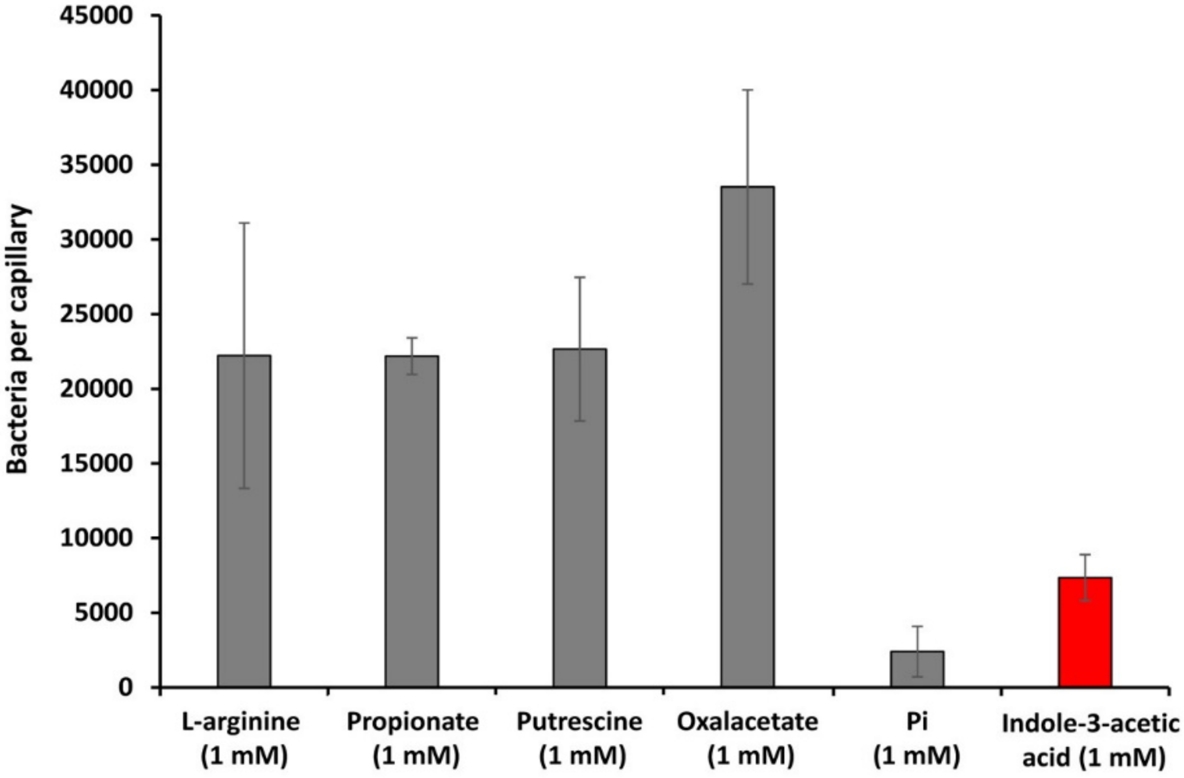

Supplementary Figure S5. Chemotactic responses of *Pseudomonas putida* 1290 to different chemoeffectors. Data are means and standard deviations from three independent experiments conducted in triplicate.

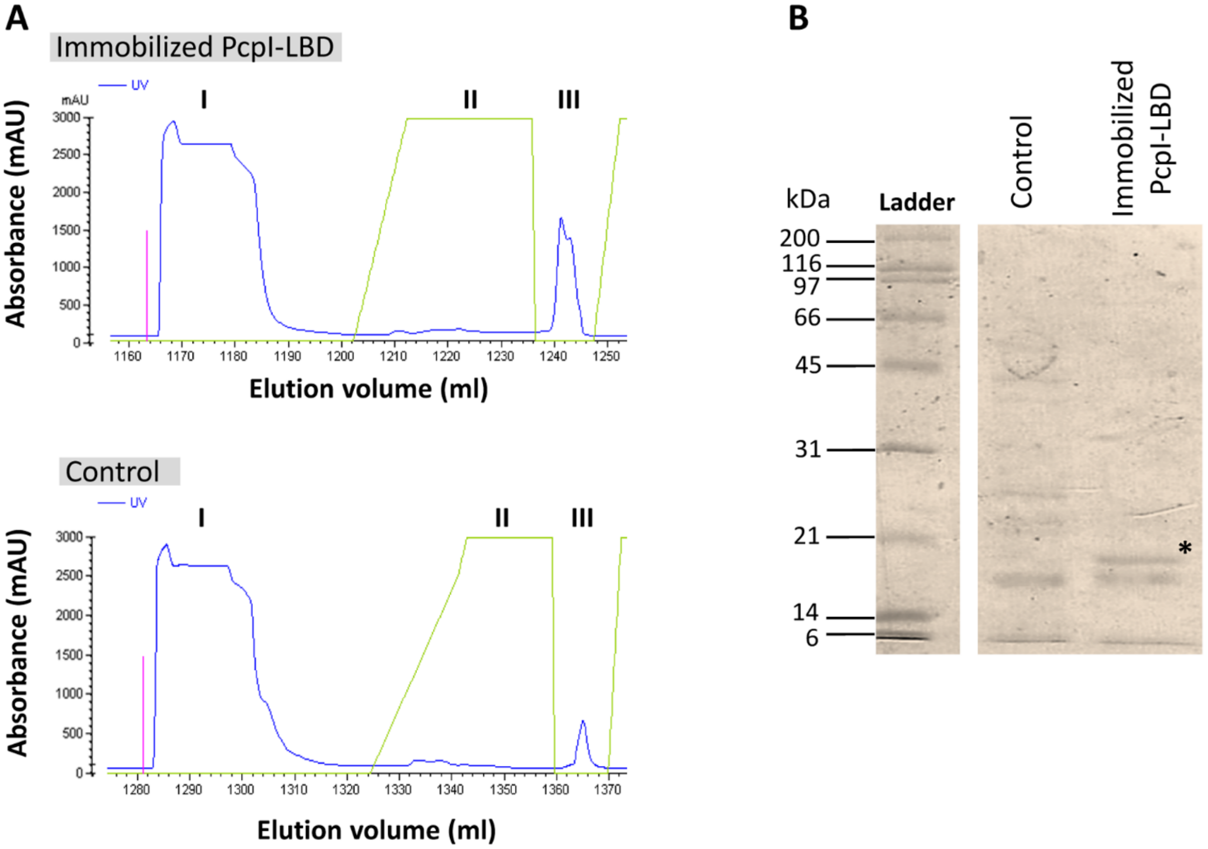

Supplementary Figure S6. Pull-down experiment on immobilized PcpI-LBD. A, FPLC (fast protein liquid chromatography) chromatogram profiles showing: (I) the injection of *Pseudomonas putida* 1290 protein extracts onto a HisTrap column without (negative control) or with immobilized PcpI-LBD; (II) protein elution using a guanidine hydrochloride gradient; and (III) PcpI-LBD protein release using a gradient of imidazole. **B,** SDS-PAGE gel of the pull-down experiment on immobilized PcpI-LBD. Shown are proteins eluted using a 0–6 M guanidine hydrochloride gradient in the control experiment (i.e. no PcpI-LBD immobilized on the column) and when PcpI-LBD was immobilized on the column. The asterisk indicates the protein band that was excised and subjected to analysis by MALDI-TOF mass spectrometry. This protein was identified as the peptidyl-prolyl *cis-trans* isomerase (PPIase) E6B08_RS26110 and regarded as an artefact of the experiment. PPIases assist protein folding by catalyzing the *cis-trans* isomerization of peptide bonds preceding prolyl residues (Göthel *et al*., 1999).

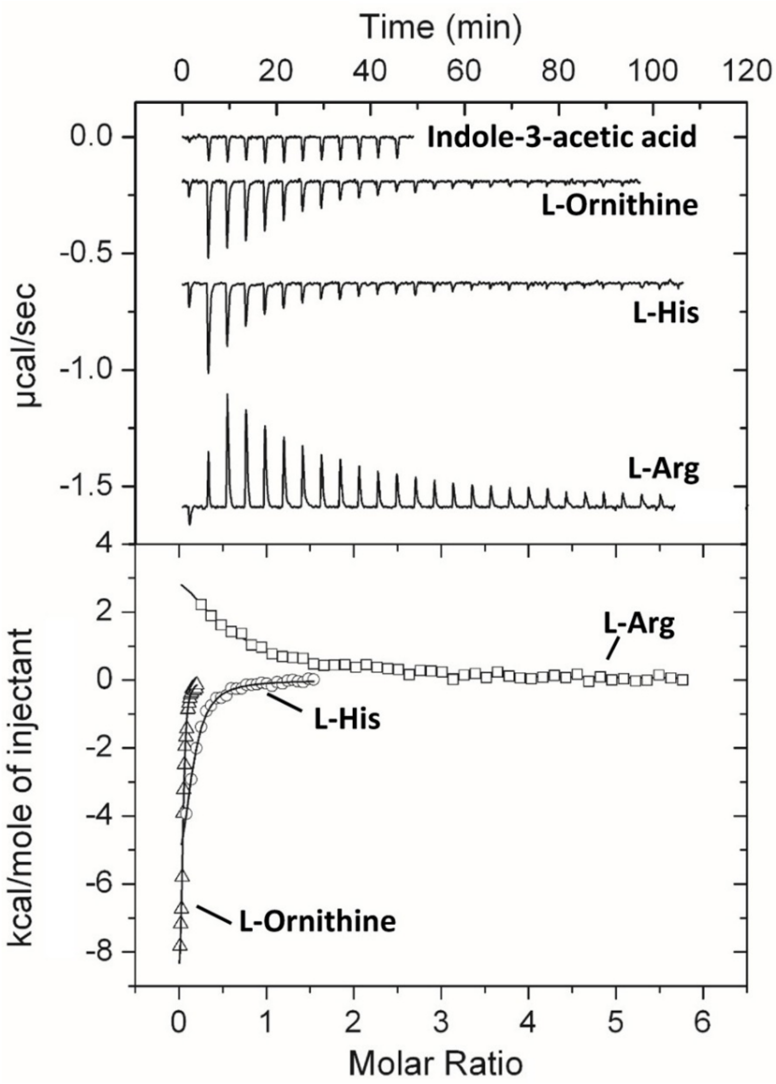

Supplementary Figure S7. The solute binding protein E6B08_RS28125 binds L-arginine, L-histidine and L-ornithine. Microcalorimetric titrations of E6B08_RS28125 with indole-3-acetic acid, L-Arg, L-His and L-ornithine. Upper graphs show raw titration data, while lower graphs show integrated corrected peak areas of the titration data fit using the “one binding site model.” The derived thermodynamic parameters are provided in Suppl. Table S1.

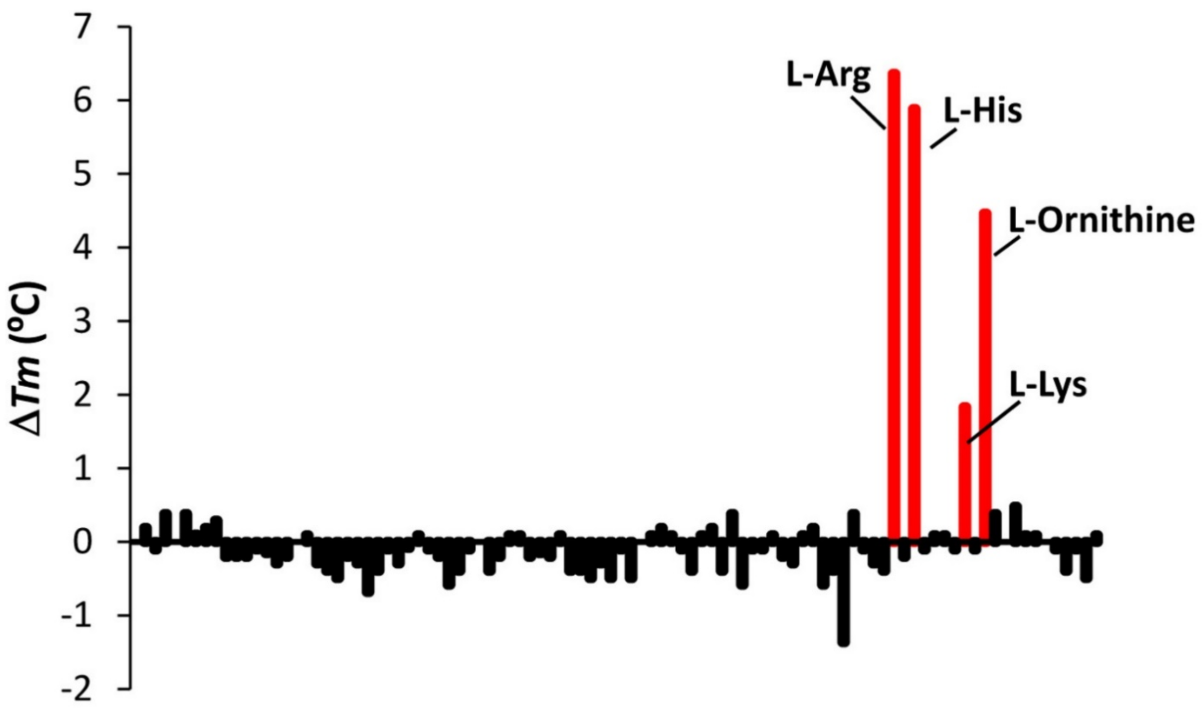

Supplementary Figure S8. Differential scanning fluorimetry-based thermal shift assays to identify E6B08_RS28125 ligands. Differential scanning fluorimetry thermal shift assays of *P. putida* 1290 E6B08_RS28125 using the 95 compounds present in the compound array PM2A (Biolog) that can serve as carbon sources. Shown are the changes in the midpoint of protein unfolding transitions (Tm) with respect to the ligand-free protein.

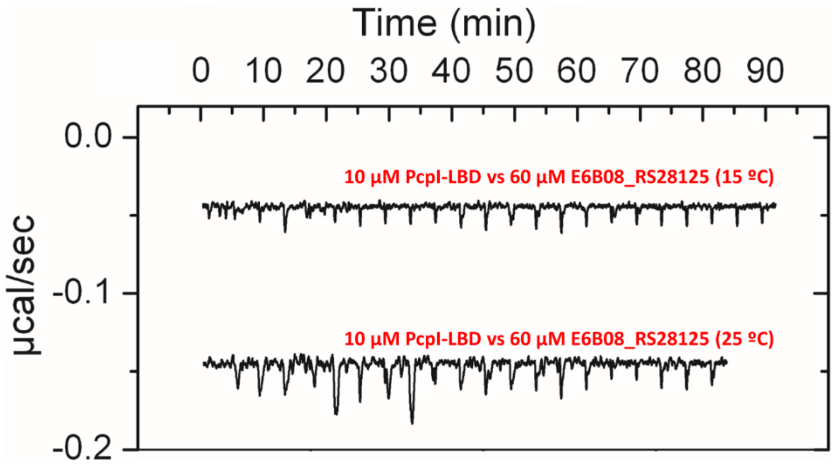

Supplementary Figure S9. Absence of interaction between PcpI-LBD and E6B08_RS28125. Microcalorimetric titrations of 10 µM PcpI-LBD with 60 µM E6B08_RS28125. To conduct the assays, both proteins were dialyzed in buffer D (5 mM Tris, 5 mM Pipes, 5 mM Mes, 10% glycerol (vol/vol), 150 mM NaCl, pH 8). The experiments were conducted at two different temperatures (e.g. 15 ºC and 25 ºC) to verify whether potential endothermic and exothermic contributions may cancel out each other at a given analysis temperature. In all cases, the injection volume was of 6.0 µl.

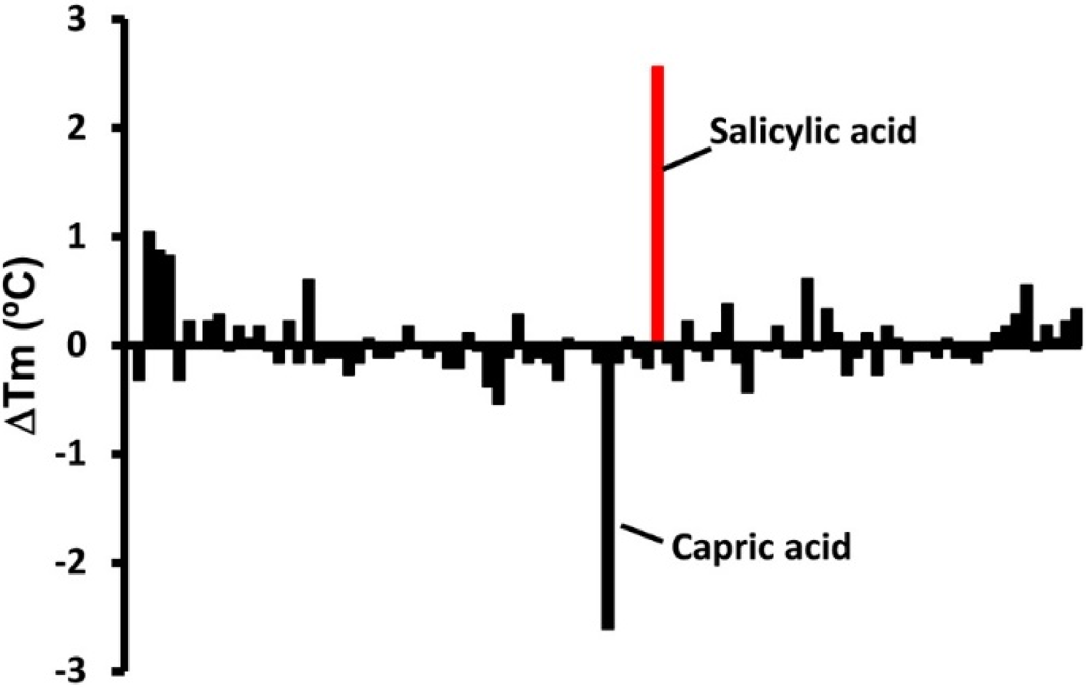

Supplementary Figure S10. Differential scanning fluorimetry based thermal shift assays to identify PcpI ligands. Shown are midpoint of protein unfolding transitions (Tm) changes for each of the 95 compounds present in the Biolog PM2A compound array of carbon sources with respect to the Tm of the ligand-free protein (39.6 °C). Microcalorimetric titrations with capric acid revealed no binding.

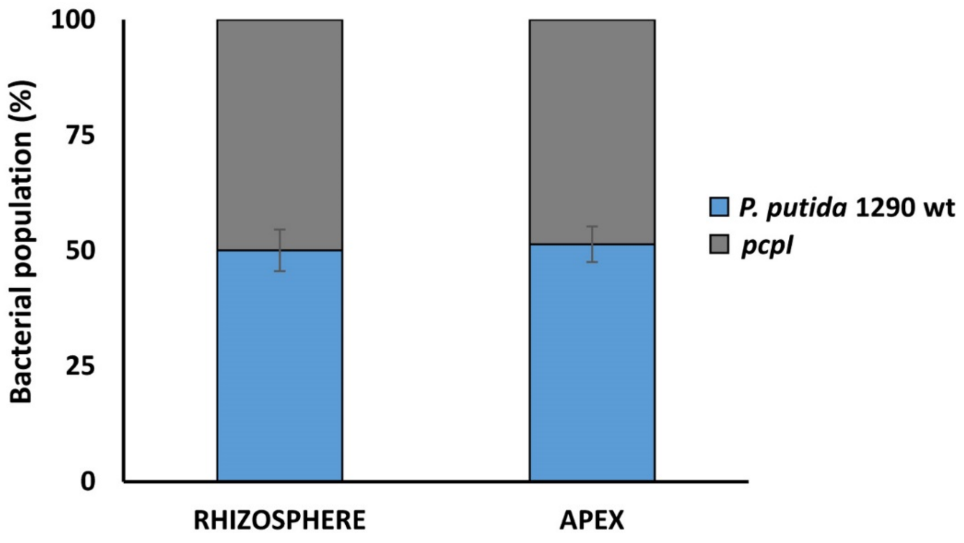

Supplementary Figure S11. Competitive root colonization of *Pseudomonas putida* 1290 and a mutant defective in *pcpI*. The figure represents the percentage of bacteria recovered either from the rhizosphere or root tips of maize (*Zea mays*) plants. Percentage of the wild type and mutant strain in the initial inoculum was 50 ± 1%. Data are the means and standard deviations of eight plants after 10 days of colonization.

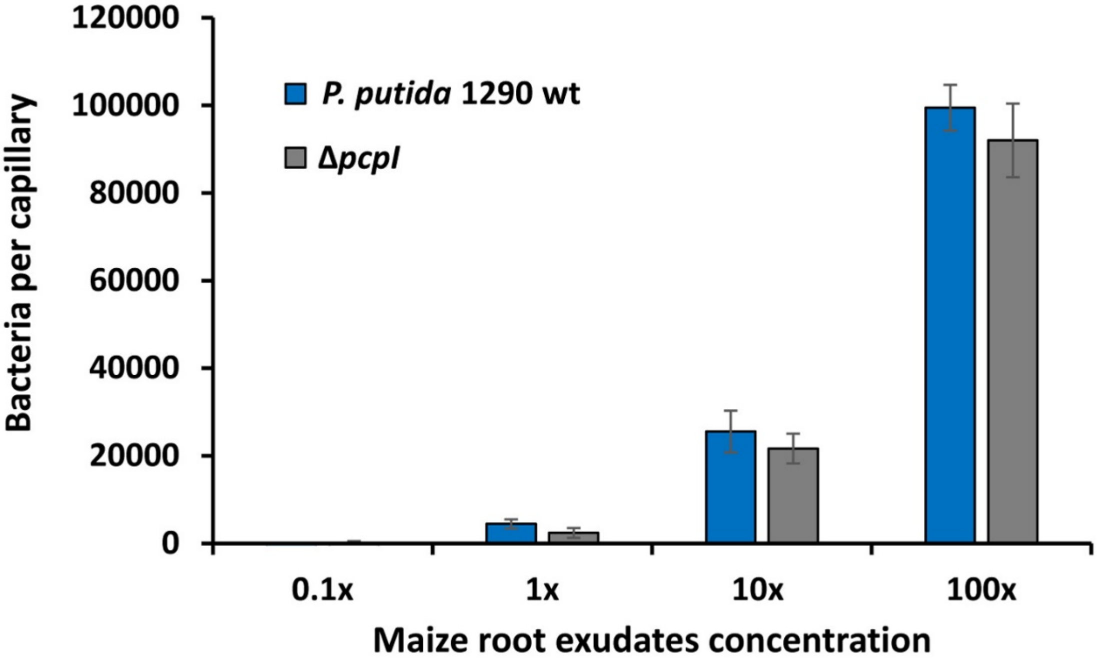

Supplementary Figure S12. Quantitative capillary chemotaxis assays of *Pseudomonas putida* 1290 strains to different concentrations of maize root exudates. Data are means and standard deviations from three individual experiments conducted in triplicate.

REFERENCES

Belda, E., van Heck, R.G.A., Lopez-Sanchez, J. M., Cruveiller, S., Barbe, V., Fraser, C., *et al*. (2016) The revisited genome of *Pseudomonas putida* KT2440 enlightens its value as a robust metabolic chassis. *Environ Microbiol* **18**: 3403–3424.

Demarre, G., Guerout, A.M., Matsumoto-Mashimo, C., Rowe-Magnus, D.A., Marliere, P., and Mazel, D. (2005) A new family of mobilizable suicide plasmids based on broad host range R388 plasmid (IncW) and RP4 plasmid (IncPalpha) conjugative machineries and their cognate *Escherichia coli* host strains. *Res Microbiol* **156**: 245–255.

Dennis, J.J. and Zylstra, G.J. (1998) Plasposons: modular self-cloning minitransposon derivatives for rapid genetic analysis of gram-negative bacterial genomes. *Appl Environ Microbiol* **64**: 2710–2715.

Göthel, S.F. and Marahiel, M.A. (1999) Peptidyl-prolyl *cis-trans* isomerases, a superfamily of ubiquitous folding catalysts. *Cell Mol Life Sci* **55**: 423–436.

Herrero, M., de Lorenzo, V., and Timmis, K.N. (1990) Transposon vectors containing non-antibiotic resistance selection markers for cloning and stable chromosomal insertion of foreign genes in gram-negative bacteria. *J Bacteriol* **172**: 6557–6567.

Jeong, H., Barbe, V., Lee, C.H., Vallenet, D., Yu, D.S., Choi, S.H., et al. (2009) Genome sequences of *Escherichia coli* B strains REL606 and BL21(DE3). *J Mol Biol* **394**: 644–652.

Kaniga, K., Delor, I., and Cornelis, G.R. (1991) A wide-host-range suicide vector for improving reverse genetics in gram-negative bacteria: inactivation of the *blaA* gene of *Yersinia enterocolitica. Gene* **109**: 137–141.

Laird, T.S. and Leveau, J.H.J. (2019) Finished Genome Sequence of the Indole-3-Acetic Acid-Catabolizing Bacterium *Pseudomonas putida* 1290. *Microbiol Resour Announc* **8**: e00519-19.

Obranic, S., Babic, F., and Maravic-Vlahovicek, G. (2013) Improvement of pBBR1MCS plasmids, a very useful series of broad-host-range cloning vectors. *Plasmid* **70**: 263–267.

Stover, C.K., Pham, X.Q., Erwin, A.L., Mizoguchi, S.D., Warrener, P., Hickey, M.J., *et al*. (2000) Complete genome sequence of *Pseudomonas aeruginosa* PAO1, an opportunistic pathogen. *Nature* **406**: 959–964.

Woodcock, D.M., Crowther, P.J., Doherty, J., Jefferson, S., DeCruz, E., Noyer-Weidner, M., *et al.* (1989) Quantitative evaluation of *Escherichia coli* host strains for tolerance to cytosine methylation in plasmid and phage recombinants. *Nucleic Acids Res* **17**: 3469–3478.
